# Effect of femoral component design and quadriceps load on patellofemoral kinematics after total knee arthroplasty: an in vitro cadaveric study

**DOI:** 10.1186/s43019-026-00308-6

**Published:** 2026-02-26

**Authors:** Vera Maioli, Emanuele Diquattro, Michele Conconi, Francesco Castagnini, Francesco Traina, Nicola Sancisi, Luca Cristofolini

**Affiliations:** 1 Department of Industrial Engineering, School of Engineering and Architecture, Alma Mater Studiorum-Università di Bologna, Via Umberto Terracini 24-28, 40131 Bologna, Italy; 2 Ortopedia-Traumatologia e Chirurgia Protesica e dei Reimpianti d’anca e di Ginocchio, IRCCS Istituto Ortopedico Rizzoli, Via Pupilli 1, 40136 Bologna, Italy

**Keywords:** Total knee arthroplasty, Patellofemoral joint, Kinematic alignment, Implant design, Patellar tracking, Femoral components design, Quadriceps load

## Abstract

**Purpose:**

Patellofemoral (PF) complications are a common cause of dissatisfaction and revision following total knee arthroplasty (TKA), often linked to altered kinematics and implant design. “Patella-friendly” femoral components with a wider, funnel-shaped trochlear groove may better restore native patellar motion. This study evaluated PF kinematics both before and after TKA performed using kinematic alignment, investigating the role of implant design and quadriceps loading.

**Methods:**

In total, 12 paired fresh-frozen cadaveric lower limbs were tested before and after TKA. Within each pair, one limb received a traditional medial pivot femoral component, while the contralateral limb received a “patella-friendly” medial pivot femoral component. Native and implanted knees were tested by flexing the knee under the action of an external load applied through the quadriceps tendon, varying its magnitudes (20, 160, 280N) and directions in the frontal (neutral,±6°, ±12°) and sagittal plane (neutral, +5° anterior). Motion was captured using an eight-camera optoelectronic system.

**Results:**

In the reference condition (20N, neutral direction), neither design showed statistical differences versus native (*p* > 0.05). However, the patella excursion in varus–valgus rotation was much higher in the specimens implanted with the traditional femoral component design (35.1° versus 14.6° native) than with the patella-friendly (20.5°). Differences between the designs emerged mainly with quadriceps load variations, especially frontal direction changes, which significantly affected patellar motion in both native and implanted knees (*p* < 0.05). Overall, the patella-friendly design better reproduced native kinematics under most conditions. However, with extreme medial loading (12°), three out of six specimens implanted with the patella-friendly femoral component were untestable owing to instability, and others exhibited high lateral displacement and trochlear dysplasia. In contrast, all traditional design implants remained stable, though with greater deviation from native kinematics.

**Conclusions:**

This study provides foundational insights into PF biomechanics before and after TKA with kinematic alignment. By analyzing the interplay between implant geometry and quadriceps loading direction, it emphasizes the importance of selecting femoral components on the basis of individual patient anatomy. Our findings suggest that patella-friendly femoral components—although capable of better reproducing native motion in some cases—may not be suitable for patients with medially directed quadriceps forces or severely varus morphotypes.

**Supplementary Information:**

The online version contains supplementary material available at 10.1186/s43019-026-00308-6.

## Introduction

Patellofemoral (PF) complications remain a major concern following total knee arthroplasty (TKA), representing a substantial source of patient dissatisfaction and revision surgery. These complications—including anterior knee pain, patellar maltracking, subluxation, fracture, patellar clunk, and crepitus—have been reported in 1.4–12.8% of cases [1, 2], depending on implant design and surgical technique [3, 4].

These complications are clinically observed in both the most common surgical alignment techniques, namely, mechanical alignment (MA) and kinematic alignment (KA) [5]. MA aims to standardize the coronal alignment by positioning the femoral and tibial components such that the joint line of the prosthesis, i.e., the line tangent to the implant surfaces in the coronal plane, is orthogonal to the mechanical axis of the limb, regardless of the patient’s native anatomy [5]. In contrast, surgery performed following the KA-TKA rationale seeks to reconstruct the patient’s native, prepathological tibiofemoral joint lines in the coronal plane, thereby preserving the individual’s limb alignments and maintaining their original Q angle [5, 6]. The Q angle is a clinical measurement to approximate the direction of the quadriceps vector force (QV). It is defined as the angle formed between the lines connecting the center of the patella to the attachment site of the patellar tendon on the tibial tubercle, and the line connecting the center of the patella to the anterior superior iliac spine on the pelvis, with the knee fully extended.

By replicating the native distal femoral joint line and Q angle, KA has the potential to promote more physiological PF mechanics, including retropatellar contact pressure, contact area, and patellar kinematics. However, in vitro studies have shown no significant differences between MA and KA-TKA with the same prosthetic design [6]. Similarly, no improvements in functional outcomes [5] or revision rate [7] have been consistently reported by clinical studies. In contrast, KA may result in slightly greater lateral patellar tilt or shift [8]. This apparent lack of clinical benefit raises a key question: if KA is expected to improve knee function, why are the reported outcomes so similar to MA?

One critical limitation of KA-TKA is the widespread use of femoral components originally designed for MA surgical technique [9]. Until a few years ago, the development of the KA surgical technique focused primarily on optimizing the alignment and function of the tibiofemoral joint, with limited attention paid to the PF joint. Traditional femoral components, originally designed for MA, are optimized for implantation following an approach that standardizes joint alignment and therefore the Q angle and are typically characterized by a trochlear groove inclined at a fixed 6° from femoral mechanical axis [10]. When traditional design components are implanted to meet the KA criteria, their geometry may no longer align with the patient-specific joint anatomy achieved with this surgical technique, in particular, at the patellar level, as the position of the implants is no longer fixed relative to the mechanical axis of the femur, potentially resulting in suboptimal patellar tracking and contributing to PF complications.

In recent years, increased awareness of the association between TKA failure and PF complications has prompted the development of so-called “patella-friendly” femoral components. Compared with the traditional components, these modern designs may incorporate several geometric modifications of the femoral component, intended to improve patellar tracking and reduce extensor mechanism-related symptoms. An example of a commonly adopted feature is the lengthening of the intercondylar notch to enhance patellar engagement during flexion so as to reduce the risk of instability or patellar “jumping” to minimize impingement with the superior edge of the femoral box, a common cause of anterior knee pain and patellar clunk syndrome [11]. Other manufacturers adopted a prominent high lateral flange, which has also been introduced to serve as a containment guide, especially during early flexion when the patella begins to engage in the trochlea [12]. This feature is particularly beneficial in patients with preexisting instability or ligamentous laxity. Moreover, patella-friendly designs may also feature a laterally oriented trochlear groove, so as to accommodate also more valgus alignment with a lateralized quadriceps line of force (quadriceps vector—QV). This kind of design may offer a particular advantage when implanted following the KA rationale, since the Q angle could vary in a wider range than in MA. Improving the congruence between the direction of the QV and the patellar motion, patella-friendly design femoral components may minimize the risk of maltracking or lateral subluxation—especially in patients with increased Q angles.

A recent radiographic study has revealed the geometric mismatch between traditional femoral component designs and the restored Q angle with KA [13]. The study demonstrated that a femoral component specifically designed for KA, with a wider trochlear groove, provided better alignment with the QV and greater lateral coverage of the anterior femoral resection. In contrast, clinical evidence remains mixed. Several studies have reported minimal and statistically nonsignificant differences in anterior knee pain, patellar clunk, and clinical outcome scores between traditional femoral components and more modern patella-friendly designs in the short and mid-term [14, 15]. Conversely, at least one long-term study has demonstrated meaningful improvements in clinical scores with patella-friendly designs [12].

Despite these clinical findings, to the best of our knowledge, no in vitro studies have conclusively demonstrated whether a patella-friendly femoral component restores PF kinematics more closely to the prepathologic native knee than a traditional design implant. Furthermore, the effect of varying quadriceps loading conditions on PF kinematics post-TKA remains underexplored.

A deeper understanding of how TKA—and, specifically, femoral component design and altered quadriceps force directions—affects PF biomechanics is essential for improving functional outcomes and reducing complication rates.

The present study aims to address the following key questions:

- How does patellar kinematics change after TKA performed with KA compared with the native knee?
- How do traditional design and patella-friendly design femoral components affect PF tracking?
- What is the effect of altered quadriceps vector direction on patellar motion?
- Does a patella-friendly design implant better preserve physiological patellar tracking under different quadriceps loading conditions?

We hypothesized that patella-friendly femoral components would more closely reproduce native patellofemoral kinematics than traditional designs under variable quadriceps loading conditions.

## Materials and methods

### Specimens

A total of 12 paired fresh-frozen human cadaveric lower limbs from six donors (Table 1) were used in this study, in accordance with the ethical approval granted by the Bioethical Committee of the University of Bologna (no. 0150450 of 5 June 2023). Specimens were obtained through an international tissue donation program (Anatomy Gift Registry, Hanover, MD, USA). Inclusion criteria were: no history of lower limb fractures or surgeries, no major deformities, and a physically active lifestyle up to the time of death. Donor demographics are summarized in Table 1.

**Table 1:** Details of specimen information and implant assignment

ID specimen	ID donor	Side	Femoral component	Sex	Age at death (years)	Height (cm)	Weight (kg)	Femoral component size	Tibial component size	
1	A	Left	Traditional	M	43	180	148	5		5
2		Right	Patella-friendly					5		5
3	B	Left	Patella-friendly	F	61	165	98	4		4
4		Right	Traditional					4		4
5	C	Left	Traditional	M	67	188	154	6		5
6		Right	Patella-friendly					6		5
7	D	Right	Traditional	M	51	188	57	5		5
8		Left	Patella-friendly					5		5
9	E	Right	Traditional	M	68	173	118	4		4
10		Left	Patella-friendly					4		4
11	F	Right	Patella-friendly	F	59	170	37	3		2
12		Left	Traditional					3		2
Mean					60	176.5	108			
Standard deviation					9.2	9.1	45.4			

Each specimen was prepared and tested following a previously established protocol [16], where the kinematics in the native conditions were investigated in detail. For completeness, a brief summary of the methodology is provided here.

### Specimens’ preparation

The specimens were stored at −28 °C sealed in plastic bags when not in use and thawed at room temperature for 24 h prior to testing. During preparation and testing, hydration was maintained through regular water spraying. Skin, subcutaneous fat, and all muscles except the distal quadriceps were removed, preserving the extensor mechanism, including joint capsule, ligaments and quadriceps tendon. The quadriceps tendon was sutured to avoid tearing under controlled loading. The femurs were proximally resected, while the tibias and fibulas were distally resected. Each end was embedded in a cylindrical cement pot, ensuring consistent alignment with the anatomical axes of the femur and tibia [17]. Stainless steel screws were placed into the femur, tibia, and patella for use as landmarks.

### Experimental set-up

A custom-built rig was used to evaluate PF kinematics during passive flexion and under simulated quadriceps load. The femur was rigidly fixed, while the tibia was suspended and free to move under gravity. The rig allowed full adjustment of femoral position, ensuring alignment with the anatomical axes and minimizing unwanted torque. Controlled quadriceps tension was applied via static weights connected to the sutured tendon through ropes and pulleys. The direction of the muscle force was modulated through adjustable pulleys mounted to the rig frame. The knee extended under quadriceps tension, while the tibial flexion was induced with a pushing rod applied to the cement pot. This method constrained only flexion, allowing the remaining degrees of motion to result from joint mechanics alone.

Motion tracking was performed using a Vicon optoelectronic system (Vicon Motion Systems Ltd.; nominal accuracy 0.5 mm/0.5°), comprising six Bonita 10 cameras (1024 × 1024 pixels, 250 Hz) and two Vero cameras (2048 × 1024 pixels, 330 Hz), arranged to maximize accuracy and minimize marker occlusion [18]. Reflective marker clusters were attached to the femur, tibia, and patella. A pointer tool with three passive markers was used to digitize the screw positions and anatomical landmarks. Prior to use, the camera system was calibrated, showing a consistent world error of less than 0.07%, which reflects the residual fitting error between the observed positions of the wand and the ideal calibration model.

### Implantation

**Fig. 1:**
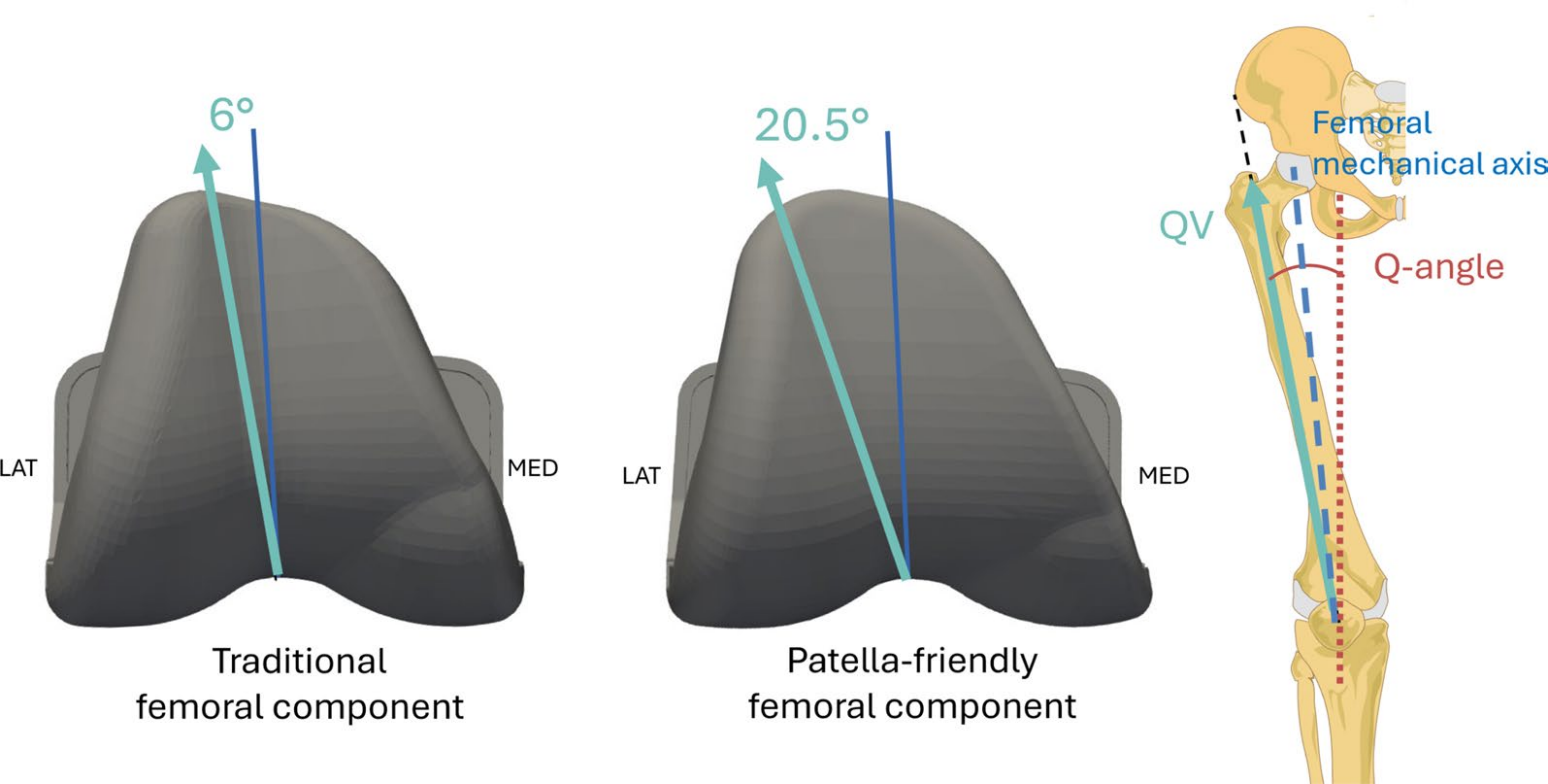
Left: traditional design femoral component with a trochlear opening at 6° to the femoral mechanical axis. Center: patella-friendly design femoral component with a trochlear opening at 20.5°. Right: the reference lines are illustrated: in black the line from the center of the patella to the ASIS, in red the line from the center of the patella to the tibial tuberosity (together forming the Q angle), and in blue the femoral mechanical axis as the line from the center of the knee joint to the center hip joint. Figshare repository (10.6084/m9.figshare.30019984)

Each specimen underwent TKA using one of the two femoral component designs from the same manufacturer (Medacta International S.A., Castel San Pietro, Switzerland): traditional design (Sphere) or patella-friendly design implant (SpheriKA). Both designs are medial-pivot type. The patella-friendly femoral component used in this study follows a design philosophy aimed at improving PF tracking by accommodating a wider range of Q angles. Compared with the traditional design, which features a trochlear opening of approximately 6° relative to the femoral mechanical axis, the patella-friendly component incorporates a substantially wider lateral opening of 20.5° (Fig. 1). Its geometry also features a more funnel-shaped trochlear groove, intended to facilitate the guided engagement of the patella during knee flexion and better support tracking, particularly under conditions of increased lateral quadriceps pull.

Knees were randomly assigned so that each donor received both implant types (one per side), with balanced distribution of right and left limbs across the two groups. Implant sizes were selected to match native femoral anatomy on the basis of preoperative computed tomography (CT) scans, and sizing was kept consistent between the two limbs of each donor (Table 1).

All the surgical procedures were carried out by an orthopedic team experienced in knee surgery. Kinematic alignment was granted, using the GMK Efficiency technique and MyKnee patient-specific cutting guides (Medacta International SA). The guides were designed from preoperative CT scans to restore the native joint lines and the individual Q angle. The only deviation from native geometry involved the tibial slope: for native slopes greater than 5°, the reconstructed slope was set to 5°; otherwise, the native slope was preserved. The patella was not resurfaced.

### Testing protocol

**Fig. 2:**
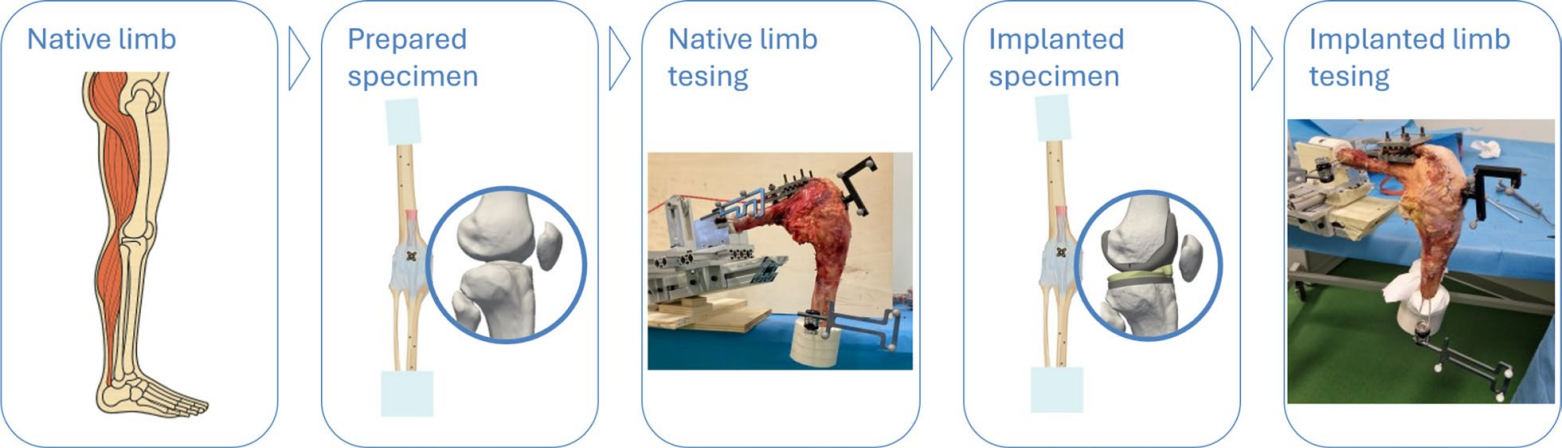
A schematic of the experimental protocol. Starting with native legs, they were prepared by removing all the soft tissues, except the joint capsule. The prepared specimens were tested. They were then implanted with TKA and tested again with the same protocol. Figshare repository (10.6084/m9.figshare.30019987)

The specimens were tested in both the native and implanted condition (Fig. 2).

**Fig. 3:**
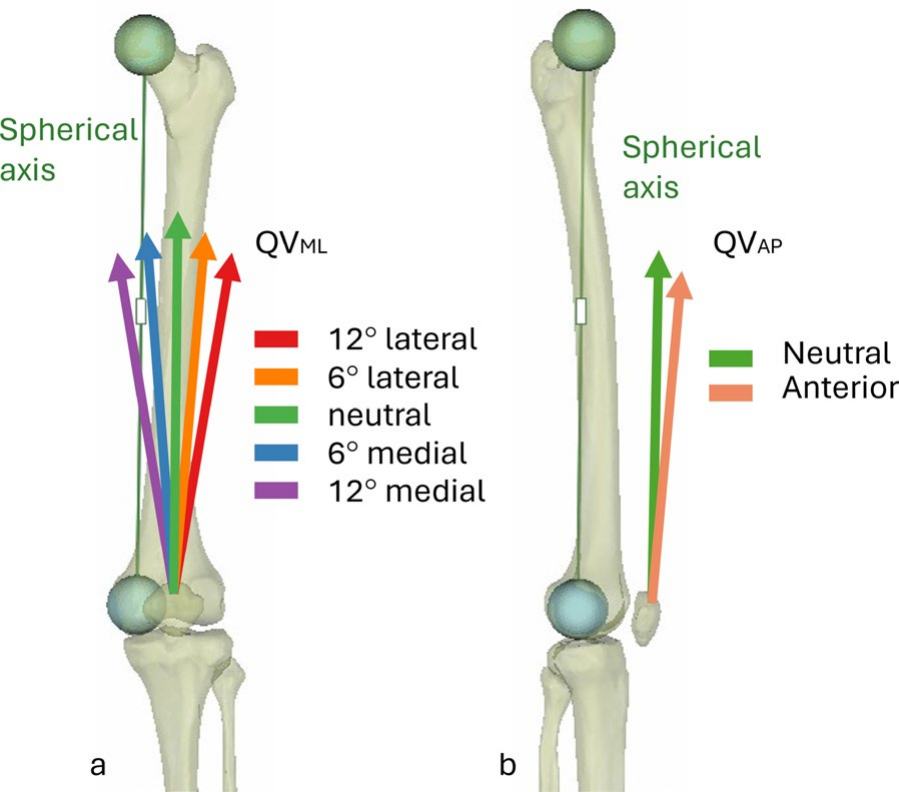
Quadriceps load variation of direction in the frontal plane (**a**) and the sagittal plane (**b**). In green, the parameters correspond to the reference conditions

Each knee was tested with ten QV directions, combining five medial–lateral (QV_ML_ in neutral, ± 6°, ± 12°) (Fig. 3a) and two anterior–posterior (QV_AP_ in neutral and + 5° anterior) (Fig. 3b) angles. The simulated variation in quadriceps direction was selected to cover the expected spectrum of Q angle variability, which can span approximately 21° (from ~6° to ~27°). Using a wide range of QV_ML_ orientations, we aimed to reproduce both physiological and pathological conditions, such as strength imbalance of the quadriceps muscle, which is a common condition after TKA [19]. This testing protocol follows the approach adopted in a previous biomechanical study evaluating patellar tracking in the native knee without TKA [16]. The neutral direction was defined as the vector parallel to a line extending from the center of the femoral head to the center of a sphere fitted on the medial femoral condyle, also referred to as the spherical axis [20]. Although the proximal femur was resected for mounting, the center of the femoral head was virtually reconstructed from the pretest CT scan of the intact limb, and the relative position of the femur and QV in the neutral direction was reconstructed with VICON three-dimensional (3D) reconstruction to ensure minimal deviation from the intended position.

Each loading direction was tested at three force magnitudes (QV_load_): 20 N (minimal preload to maintain patellar position), 160 N, and 280 N (simulating two levels of muscle contraction). Thus, a total of 30 tests were performed on each native and on each implanted specimen.

For each test, the starting position was defined as the maximal flexion reached by pushing the tibia. The end position was defined as the maximum extension reached under quadriceps loading, except for the minimal load condition (20 N), where the extension was induced. Each test included three cycles of flexion–extension, followed by a rest period of approximately 1 min.

### Imaging and data analysis

CT scans (CT, GE Discovery CT scanner, GE Healthcare, Milwaukee, WI; slice thickness = 0.625 mm, in-plane resolution = 0.781 mm) were acquired at three time points: on the entire limb before starting any specimen preparation, after preparation for testing, and after TKA implantation. The 3D models of the bone surfaces were generated using semi-automatic segmentation (Mimics v25.0, Materialise NV, Leuven, Belgium). Anatomical reference frames were defined on the segmentations obtained from the native leg, on the basis of identifiable landmarks, following established conventions [21].

**Fig. 4:**
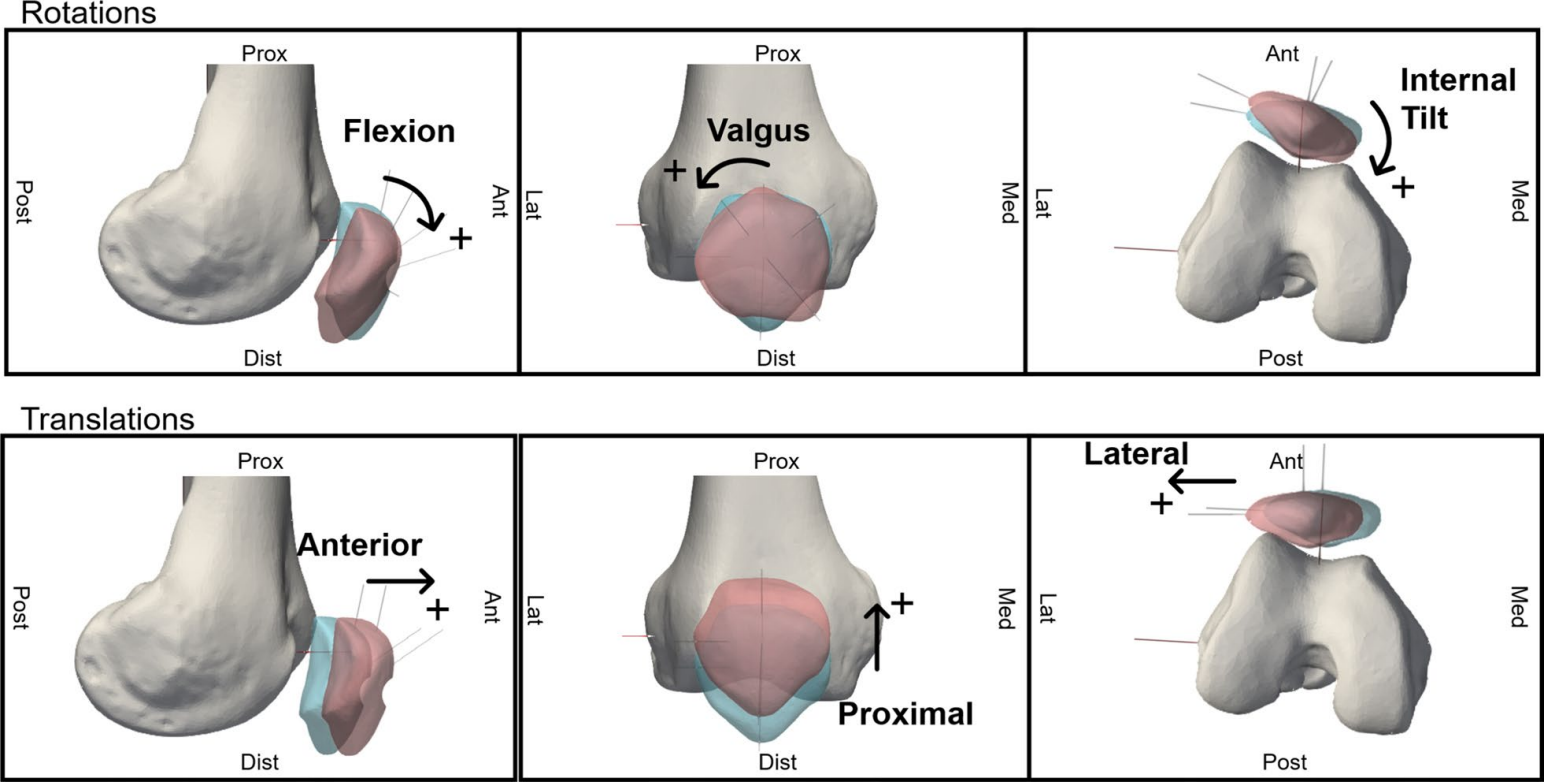
Motion components of the patellofemoral joint of a right leg. Figshare repository (10.6084/m9.figshare.30019969)

Knee joint motion was analyzed with the proprietary software of the optoelectronic system (Nexus 2.5, Vicon, Oxford, UK), which allowed for the reconstruction of marker trajectories throughout the test sequences. Marker data were transformed from the laboratory coordinate system to anatomical reference frames via a CT-based registration pipeline. Registration was performed using a point-to-surface distance minimization algorithm, both on the bone geometry and on three non-collinear fiducial markers per bone to improve accuracy. After registration, mean distances between fiducial points were measured to generate a distance map, and the registration was considered satisfactory if errors were below 0.5 mm. The complete transformation process is detailed in a previous work [16]. The relative motion of the patella with respect to the femur and of the femur with respect to the tibia were quantified in MATLAB (v2023b, MathWorks Inc.), using the Grood and Suntay convention [22] to describe motion in terms of three rotations and three translations (Fig. 4).

### Metric

Initially, the comparison of the patella behavior before and after TKA was referred to a single condition, which is considered the reference condition. This is minimal load (20N) and neutral direction in both medial–lateral and anterior–posterior directions (green arrows in Fig. 3). In this condition, patellar tracking was evaluated for all grouped specimens by comparing the native knee with the implanted patella both for the specimens implanted with traditional prostheses and with patella-friendly prostheses.

Subsequently, the influence of varying quadriceps load direction and magnitude on patellar motion was assessed for both native and implanted conditions. To minimize interspecimen variability, the effect of these perturbations was quantified relative to the reference condition of the same specimen. Specifically, in the native knee, each loaded test was compared with the native reference test, while in the implanted knee each loaded test was compared with the implanted reference test. This approach allowed us to isolate the effect of load variations independently of the interspecimen differences in native and implanted states, assessed with the previous analysis.

Finally, given that the patella-friendly design device was specifically intended to address Q angle variability, here simulated by changing QV_ML_, a focused transversal analysis was performed on the influence of QV_ML_ on PF kinematics. Comparisons were made across native knees, traditional design implants, and newer patella-friendly design implants.

### Statistical analysis

The distribution of all kinematic variables was assessed using the Shapiro–Wilk test, which indicated a deviation from normality for some degrees of knee flexion. As a result, nonparametric statistical methods were adopted.

In the reference condition, the effect of TKA on patellar kinematics was assessed separately for the two implant designs. Specimens were divided into two groups according to the femoral component implanted: traditional design and patella-friendly design. For each group, both the native and the implanted condition were available, allowing paired comparisons of pre- and postimplantation kinematics within the same design. Statistical analysis was performed using the Wilcoxon signed-rank test at 1° knee flexion intervals, comparing native versus implanted knees within each group. To account for multiple comparisons, *p*-values were adjusted using the Bonferroni correction, and statistical significance was set at *p* < 0.05.

To evaluate the effects of quadriceps loading parameters on PF kinematics, we analyzed separately the influence of QV_ML_, QV_load_, and QV_AP_. For each parameter, data were grouped according to its levels while keeping the other parameters unstratified. This procedure was performed both in the native and implanted condition. To minimize interspecimen variability, for each specimen and test the kinematic curve was normalized by subtracting the corresponding specimen-specific reference condition (20 N load, neutral direction). Statistical analysis was then conducted using the Kruskal–Wallis test at 1° knee flexion intervals, with Bonferroni correction (*p* < 0.05), comparing each test condition to its reference.

To investigate the effect of femoral component design on patellar tracking under different quadriceps loading directions, a linear mixed-effects model was used. Specimens were grouped according to the implanted femoral component (traditional design versus patella-friendly design), and the statistical model assessed differences in PF kinematics between the native and implanted conditions (native as baseline), accounting for quadriceps vector direction (QV_ML_) as a fixed effect (neutral direction as baseline) and specimen identity as a random effect. This approach allowed for the inclusion of repeated measures within specimens and provided a robust evaluation of the interaction between prosthetic design and load direction. The analysis was conducted for all the motion components at 1° intervals of knee flexion. The results were considered significant for *p* < 0.05.

A summary of the statistical analysis used is reported in Table 2. All analyses were conducted using R (version 4.0.1, http://www.r-project.org).

**Table 2:** Overview of statistical analyses used for each comparison

Research question	Comparison	Statistical test	Notes/Corrections
Effect of TKA on PF kinematics in the reference condition	Native versus implanted (within design)	Wilcoxon signed-rank test	Bonferroni correction; *p* < 0.05
Effect of quadriceps parameters (QV_ML_, QV_AP_, QV_load_)	Each parameter level versus reference (QV_ML_ = neutral, QV_AP_ = neutral, QV_load_ = 20N)	Kruskal–Wallis test	Specimen-normalized; Bonferroni correction; *p* < 0.05
Effect of QV_ML_ with different implant designs	Native kinematic versus implanted kinematic with traditional femoral design versus implanted kinematic with patella-friendly femoral design	Linear mixed-effect model	Specimen as random effect; *p* < 0.05

## Results

Native knee tests were performed correctly on all specimens except specimens 11 and 12, which belonged to the same donor. Tests with QV_ML_ at 12° medial were not performed for these two specimens owing to the risk of damage to the extensor mechanism. The same issue occurred post-TKA in specimens 6, 10, and 11, all implanted with the patella-friendly design.

Specimens that could not complete the 12° medial QV_ML_ tests were excluded only from the analyses of the specific tests that were unperformed. All other test conditions from these specimens were included in the analysis.

In the following presentation of results and discussion, we will focus solely on PF kinematics. Only the graphs of the most significant motion components will be shown, while the complete analysis of all motion components can be found in Supplementary Material 1. Details on tibiofemoral kinematics are provided in Supplementary Material 2.

### Comparison between native and implanted knees at the reference condition

**Fig. 5:**
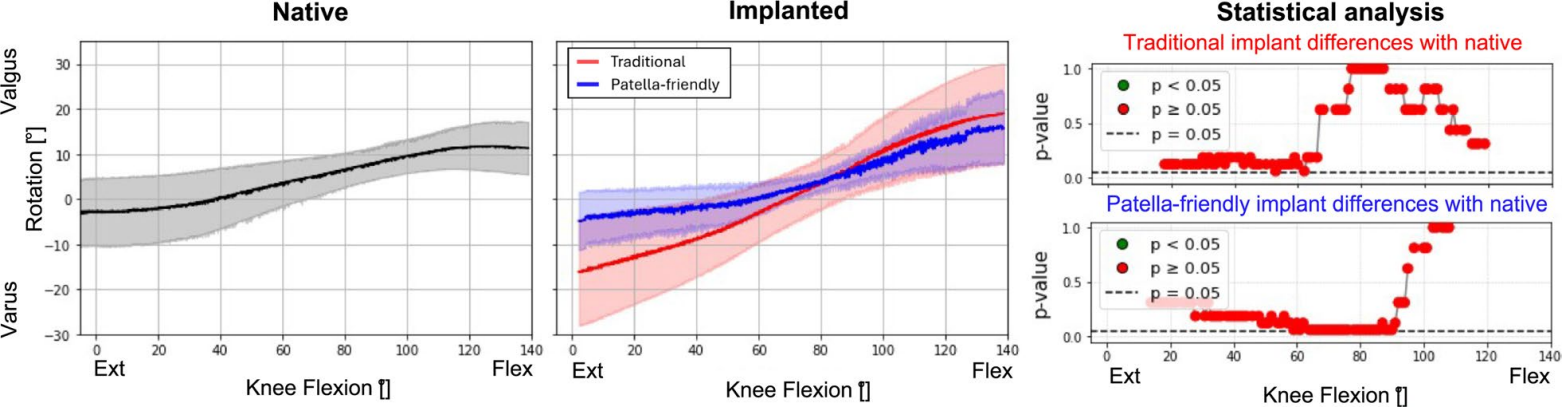
varus–valgus rotation of the patellofemoral joint before (first column) and after (second column) TKA, grouped according to the design of the femoral component. The third column represents the results of the statistical analysis of the differences between before and after TKA on the top for the traditional implant design and on the bottom for the patella-friendly design (in green the significant values, in red the nonsignificant values, threshold value 0.05). Data include 12 specimens in the native condition and 6 per design in the implanted condition

**Fig. 6:**
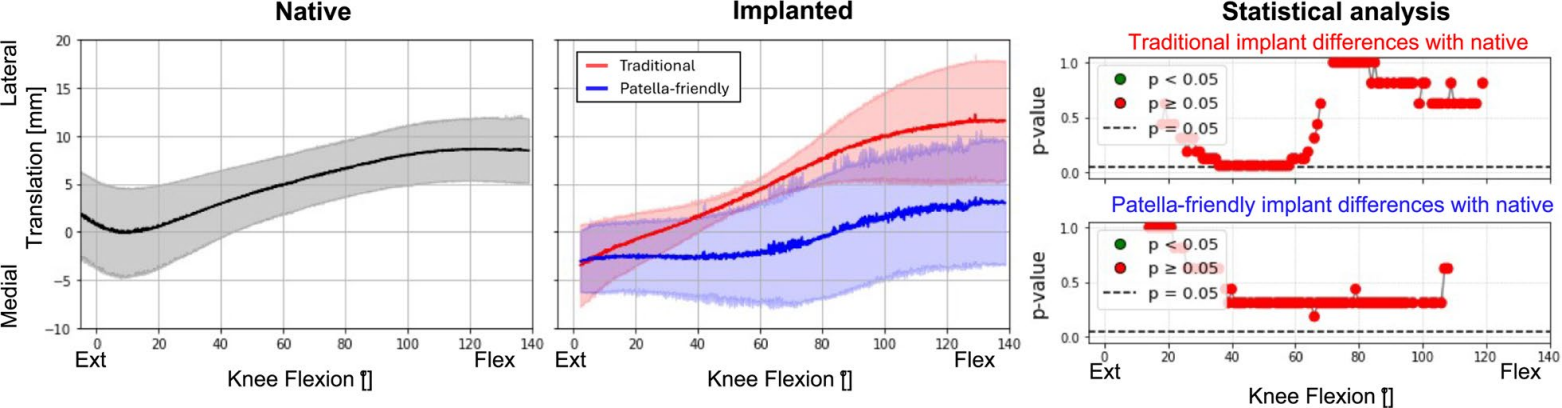
Medial–lateral translation of the patellofemoral joint before (first column) and after (second column) TKA, grouped according to the design of the femoral component. The third column represents the results of the statistical analysis of the differences between before and after TKA on the top for the traditional implant design and on the bottom for the patella-friendly design (in green the significant values, in red the nonsignificant values, threshold value 0.05). Data include 12 specimens in the native condition and 6 per design in the implanted condition

**Fig. 7:**
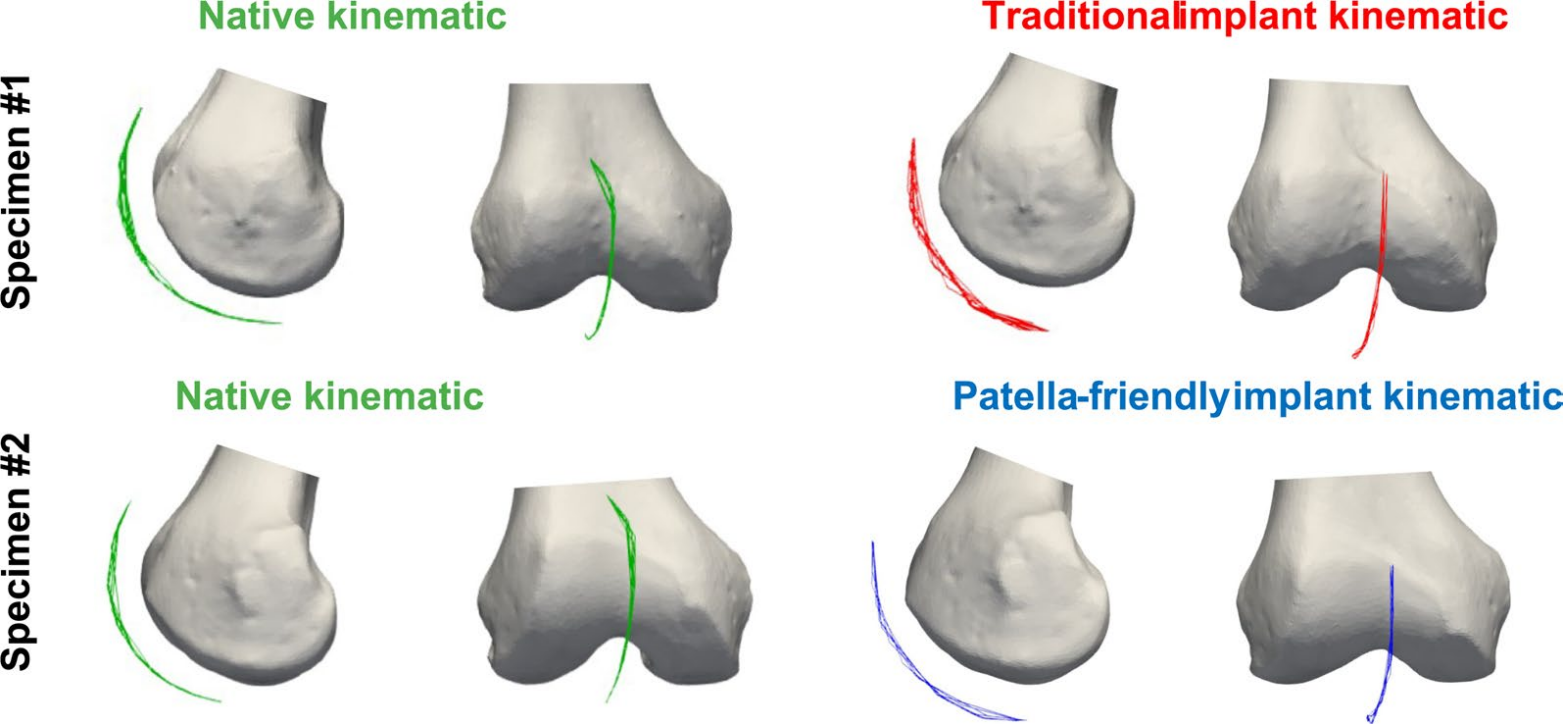
Trajectory of the centroid of the patella during testing in the reference condition in two specimens (right and left leg from the same donor) before and after TKA implantation. The top specimen (left leg, image was mirrored to make it easier to compare) was implanted with the traditional design prosthesis; the bottom specimen (right leg) was implanted with the patella-friendly design prosthesis. The trajectory of the patella is always represented for a knee flexion from 0° to 120°

Analysis at the reference condition (20 N quadriceps force, neutral in the frontal and sagittal planes) revealed no significant differences in PF kinematics between the native and implanted knees for both the femoral component designs. However, the curve’s trends showed several interesting features, especially in varus–valgus rotation (Fig. 5), medial–lateral translation (Fig. 6) and anterior–posterior translation (Fig. 7) of the patella. The main results are summarized in Table 3.

**Table 3:** Summary of the results obtained in the reference condition

PF kinematic motion component	Traditional femoral component	Patella-friendly femoral component
	Patellar excursion	Patellar excursion
Flexion–extension rotation	Similar to native	Similar to native
Varus–valgus rotation	Higher patellar excursion than native	Similar to native
Internal–external tilt	Similar to native	Similar to native
Anterior–posterior translation	Similar to native	Similar to native
Proximal–distal translation	Similar to native	Similar to native
Medial–lateral translation	Greater overall translation; more lateral patella than native in full flexion	Similar range of motion; patella more medially translated than native throughout the flexion knee

**Fig. 8:**
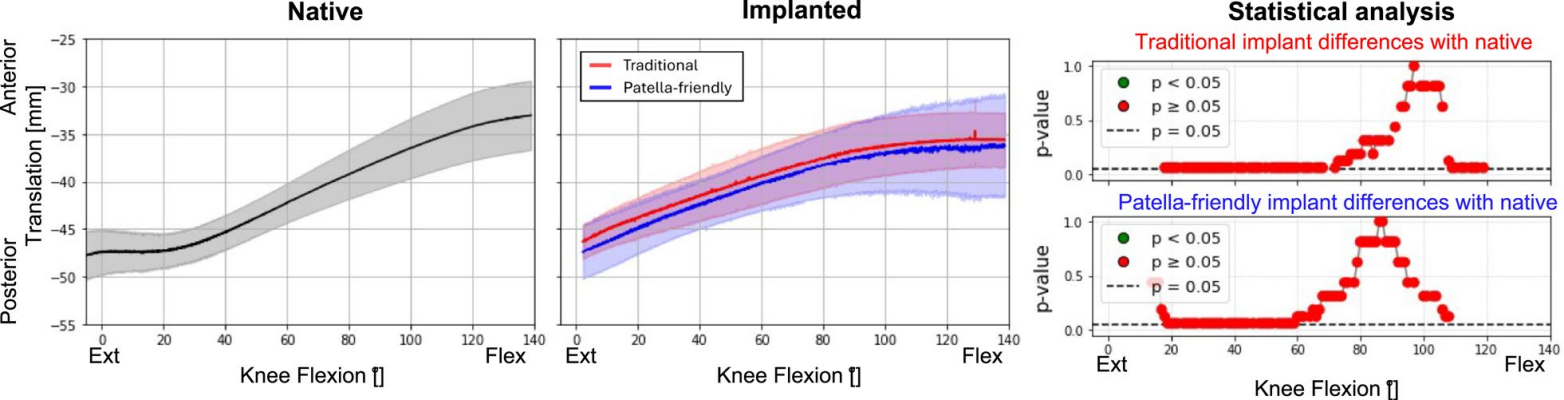
Anterior–posterior translation of the patellofemoral joint before (first column) and after (second column) TKA, grouped according to the design of the femoral component. The third column represents the results of the statistical analysis of the differences between before and after TKA, on the top for the traditional implant design and on the bottom for the patella-friendly design (in green the significant values, in red the nonsignificant values, threshold value 0.05). Data include 12 specimens in the native condition and 6 per design in the implanted condition

Notably, the characteristic early flexion medial shift of the native patella was reduced with the patella-friendly design and was completely absent with the traditional design (Fig. 6). This altered early tracking was also evident in the patellar centroid trajectory (Fig. 7). Anterior–posterior translation showed a similar trend: native knees translated minimally in early flexion, whereas implanted knees began translating immediately (Fig. 8). Although these differences did not reach statistical significance owing to the relatively high interspecimen variability, the observed pattern suggests a potentially relevant alteration of early patellar tracking following TKA.

### Influence of quadriceps loading parameters

The effect of quadriceps loading parameters on native PF kinematics was studied in detail in a previous work [16], and it is used here as a comparison for the discussions.

**Fig. 9:**
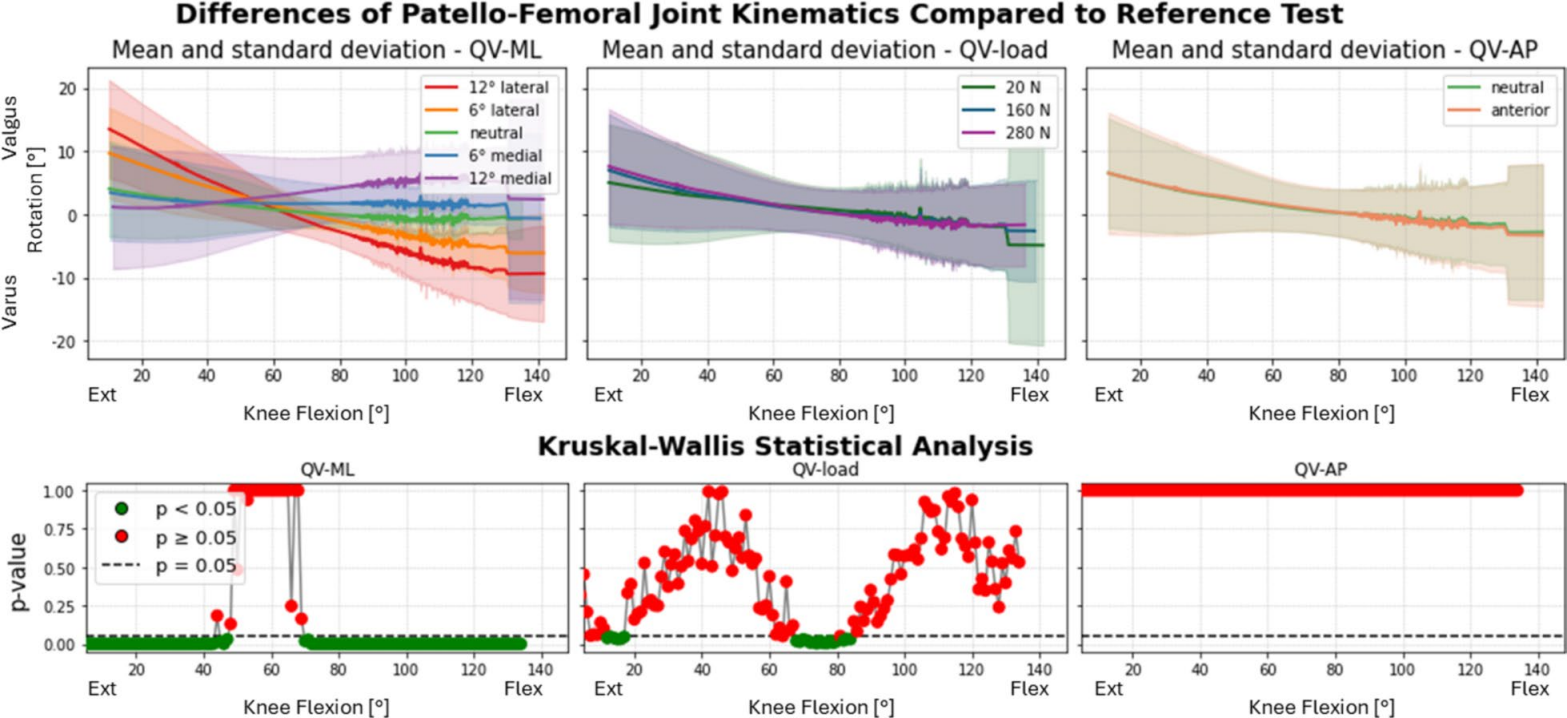
Varus–valgus rotation of the patella after TKA. Top: absolute values as a function of knee flexion angle (the median and standard deviation between 12 specimens are plotted). Center: differences of all tests compared with the reference test (QV_load_ = 20 N, QV_ML_ = neutral, QV_AP_ = neutral). Left: the difference as a function of QV_ML_, middle QV_load_, right QV_AP_. Bottom: significance of the differences plotted at the center. The *p*-value trend is plotted for the three parameters (left QV_ML_, middle QV_load_, right QV_AP_); the threshold for significance (*p* = 0.05) is marked as a dashed line. The significant values are represented in green, the nonsignificant ones in red

**Fig. 10:**
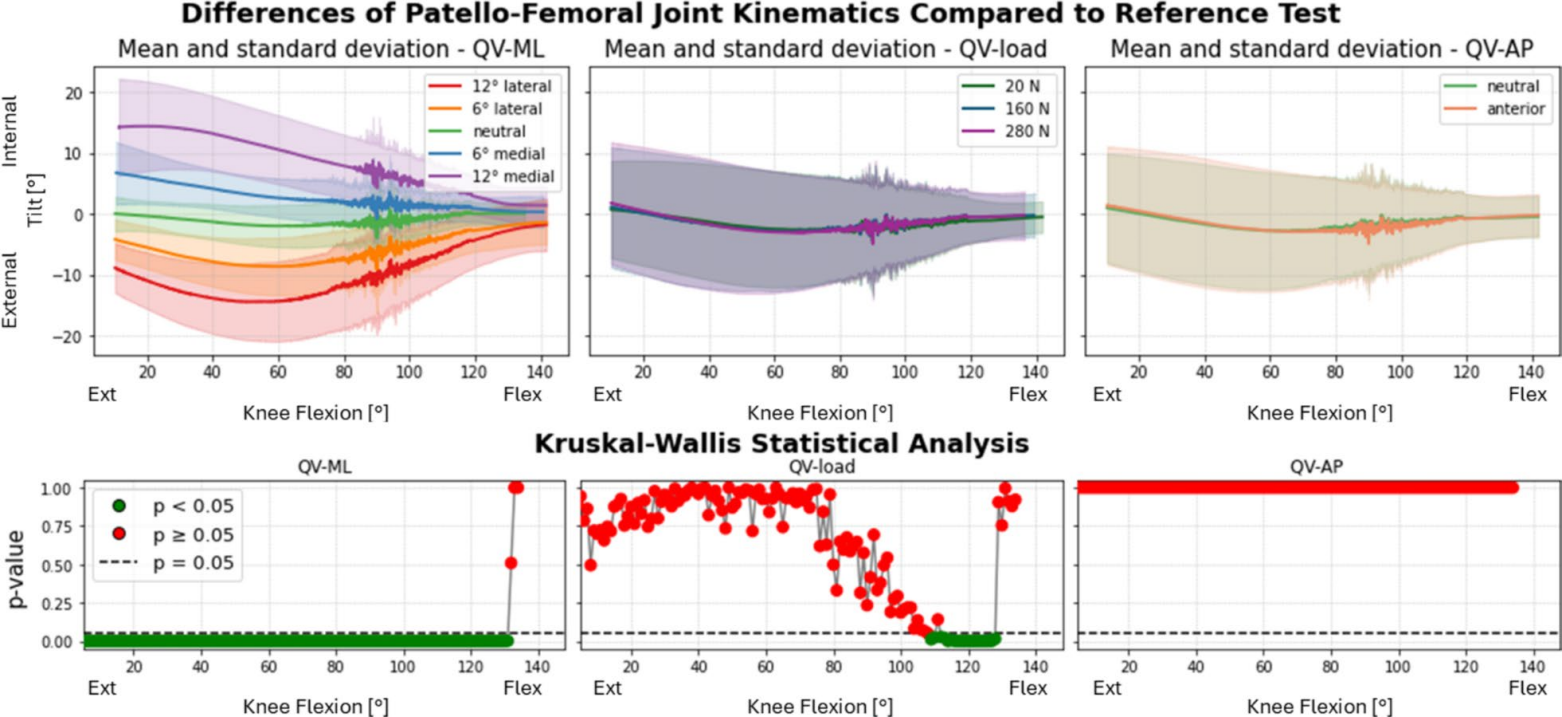
Internal–external tilt of the patella after TKA. Top: absolute values as a function of knee flexion angle (the median and standard deviation between 12 specimens are plotted). Center: differences of all tests compared with the reference test (QV_load_ = 20 N, QV_ML_ = neutral, QV_AP_ = neutral). Left shows the difference as a function of QV_ML_, middle QV_load_, right QV_AP_. Bottom: significance of the differences plotted at the center. The *p*-value trend is plotted for the three parameters (left QV_ML_, middle QV_load_, right QV_AP_); the threshold for significance (*p* = 0.05) is marked as a dashed line. The significant values are represented in green, the nonsignificant ones in red

**Fig. 11:**
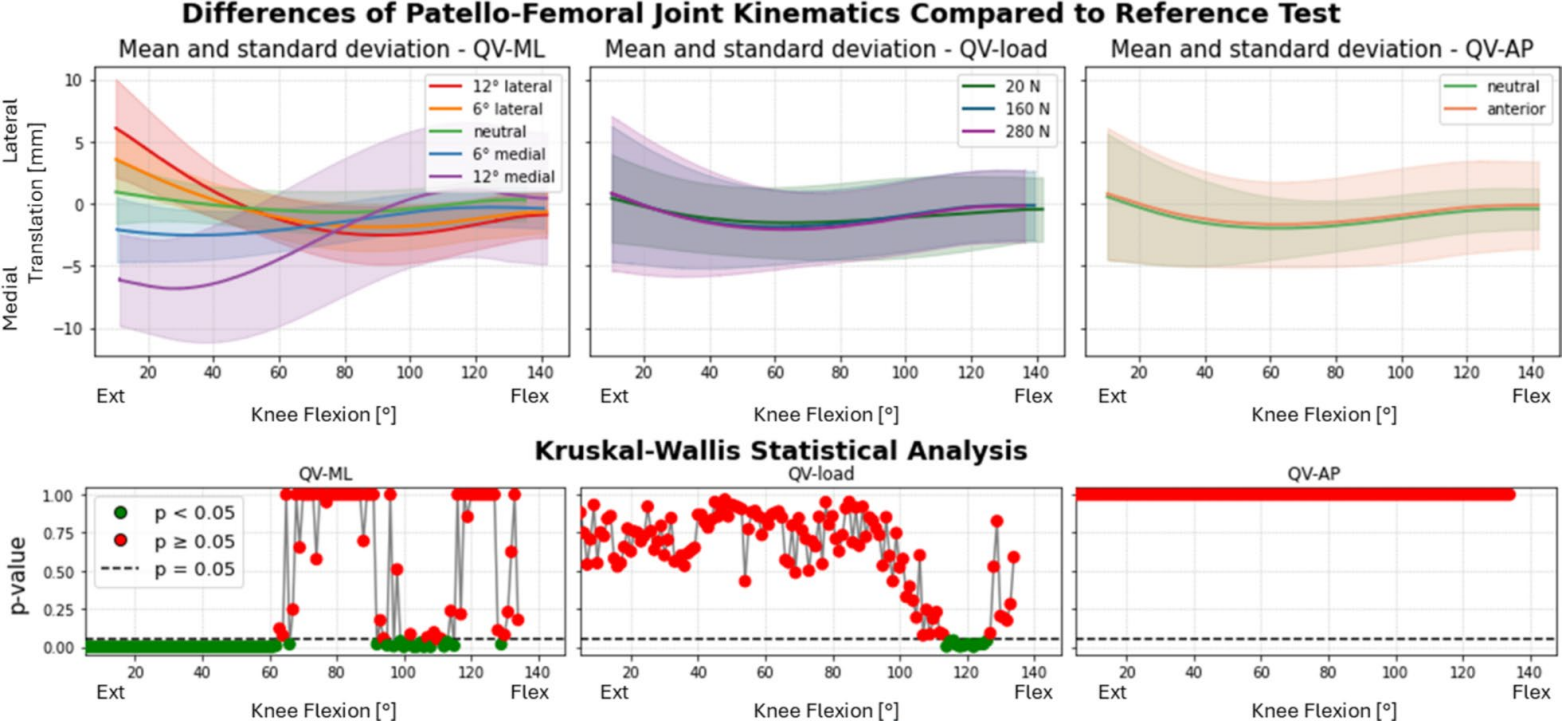
Medial–lateral translation of the patella after TKA. Top: absolute values as a function of knee flexion angle (the median and standard deviation between 12 specimens are plotted). Center: differences of all tests compared with the reference test (QV_load_ = 20 N, QV_ML_ = neutral, QV_AP_ = neutral). Left shows the difference as a function of QV_ML_, middle QV_load_, right QV_AP_. Bottom: significance of the differences plotted at the center. The *p*-value trend is plotted for the three parameters (left QV_ML_, middle QV_load_, right QV_AP_); the threshold for significance (*p* = 0.05) is marked as a dashed line. The significant values are represented in green, the nonsignificant ones in red

In the knees implanted with both the traditional design and the patella-friendly design components, the medial–lateral direction of the quadriceps vector (QV_ML_) significantly affected varus–valgus rotation (Fig. 9), internal–external rotation (Fig. 10), and medial–lateral translation (Fig. 11) of the patella over the full flexion range.

In particular, with the leg extended, the patella pulled by the lateral QV_ML_ is in a valgus position, externally tilted and more laterally translated; with the leg flexed, the patella is in a neutral tilt and varus position. Conversely, when the QV_ML_ acts in the medial direction, the patella shifts from a varus, medial position with internal tilt to a valgus position with a neutral tilt. The varus–valgus inversion consistently occurred around 60°, where QV_ML_ ceased to influence patellar behavior, which became more dependent on QV_load_. Around the same angle, all the medial–lateral translations become smaller and independent on the QV_ML_, apart from the 12° medial direction. QV_AP_ and QV_load_ had small or null but statistically significant effects on both rotations and medial–lateral translation.

The trend of all the motion components is detailed in Supplementary Material 1. Furthermore, in Supplementary Material 3, the absolute PF kinematics (without subtracting the reference test in neutral condition) is reported for all tests performed before and after TKA with the traditional design component, grouped by parameters for one specimen (specimen 1). In Supplementary Material 4, the same results are reported for specimen 2, from the same donor but implanted with the patella-friendly design component.

### Effect of the quadriceps vector direction on kinematic fidelity to native motion

The greater influence of QV_ML_ justified the focused transversal analysis conducted to assess differences between native and implant PF kinematics when the direction of the quadriceps force varied in the medial–lateral direction.

Overall, PF kinematics was significantly affected by the presence of TKA, with effects varying according to implant design and QV_ML_. The three motion components most affected were medial–lateral translation, varus–valgus rotation, and internal–external tilt. The patella-friendly design better mimicked native PF kinematics compared with the traditional design across all motion components (Table 4).

**Table 4:** Effect of femoral component design on patellar kinematics and quadriceps action across flexion

PF kinematic motion component	Traditional femoral component	Patella-friendly femoral component
Varus–valgus rotation	Alters patellar rotation throughout knee flexion; Modifies the relationship between patellar motion and quadriceps action at the knee flexed	Alters patellar motion throughout knee flexion, but to a lesser extent; Minimal interference with quadriceps action
Internal–external tilt	Influences tilt throughout knee flexion; Alters the quadriceps effect on the patella, especially for medial-directed quadriceps	No significant alteration of tilt; Minimal effect on quadriceps action
Medial–lateral translation	Significantly affects patellar translation throughout flexion except at full extension; Modifies the quadriceps effect when the knee is flexed	Significant effect only for extreme medial quadriceps direction; Otherwise, minimal effect; interaction with quadriceps only for extreme medial direction

**Fig. 12:**
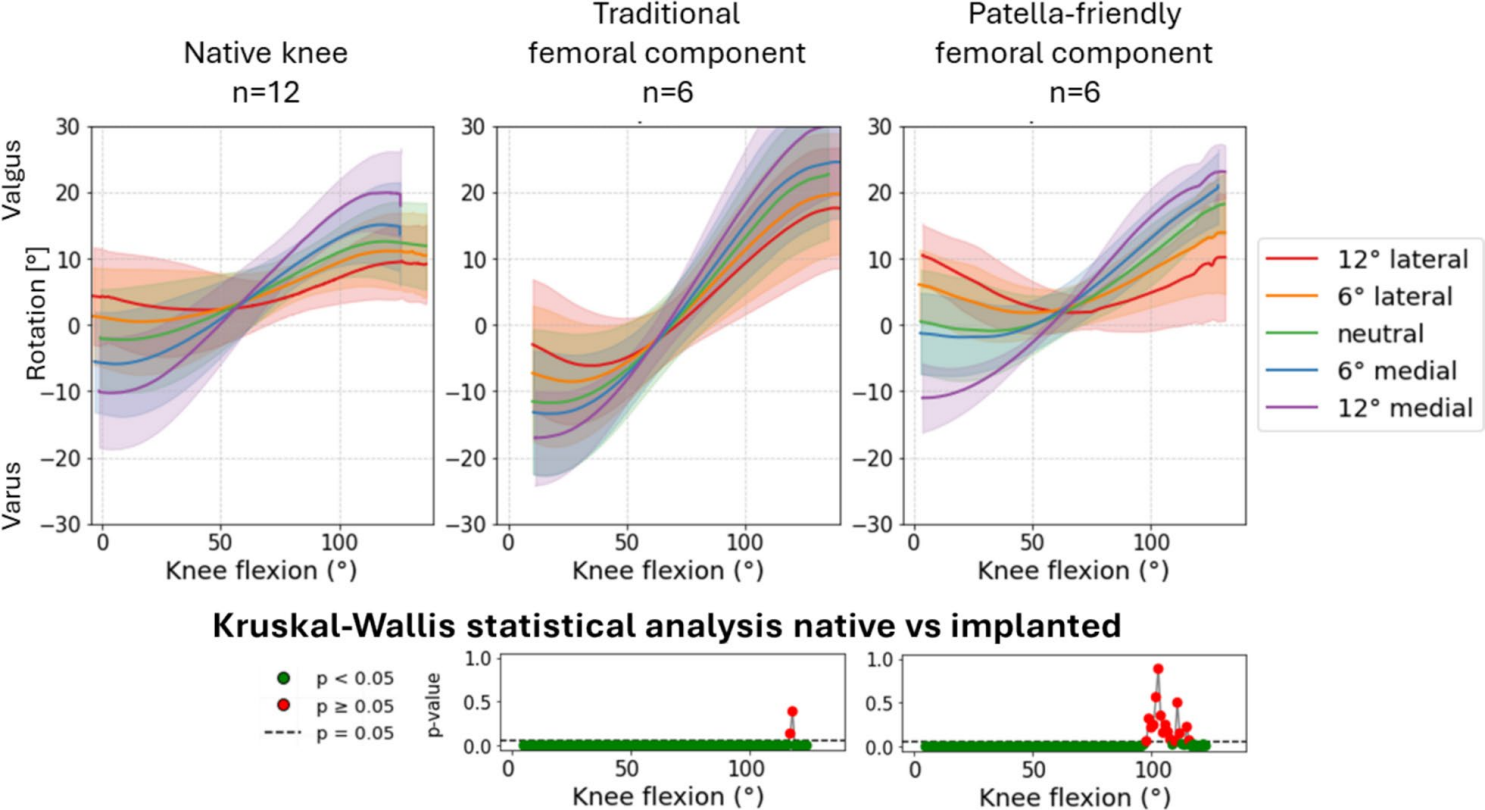
Varus–valgus rotation as a function of medial–lateral direction of the quadriceps force in native limbs (left), limbs implanted with traditional design TKA (center), and limbs implanted with patella-friendly design TKA (right). Bottom: the results of the mixed effect models statistical analysis for the presence of TKA versus native grouped for the traditional and patella-friendly design femoral component. The significant values are represented in green and the nonsignificant ones in red, and the threshold for significance (*p* = 0.05) is marked as a dashed line

An analysis of patellar varus–valgus rotation (Fig. 12) confirmed that traditional design implants produced a greater rotational excursions than patella-friendly design, deviating more from the native knees behavior.

More generally, the traditional design resulted in significant differences from the native kinematics throughout knee flexion–extension cycles (*p* < 0.001), as was considered the direction of QV alone. In contrast, the interaction between TKA condition (native versus implanted) and QV direction was significant (*p* < 0.05) for all directions, but not at full flexion. In the mixed-model analysis, the “interaction” terms tested whether the effect of QV direction on patellar motion differed between native and implanted knees. A significant interaction, therefore, indicates that the implant condition modified the way in which QV direction influenced varus–valgus kinematics, rather than simply adding a constant offset. This indicates that the influence of QV direction on varus–valgus motion was different in native and implanted knees, but at high flexion angles, the effect of the implant alone was predominant.

In the patella-friendly design group, the range of motion was generally more similar to native. However, the presence of TKA alone produced a significant alteration of PF kinematics (*p* < 0.05). Again, the direction of QV alone strongly influenced the motion throughout the knee flexion (*p* < 0.001), while few interaction terms reached significance.

**Fig. 13:**
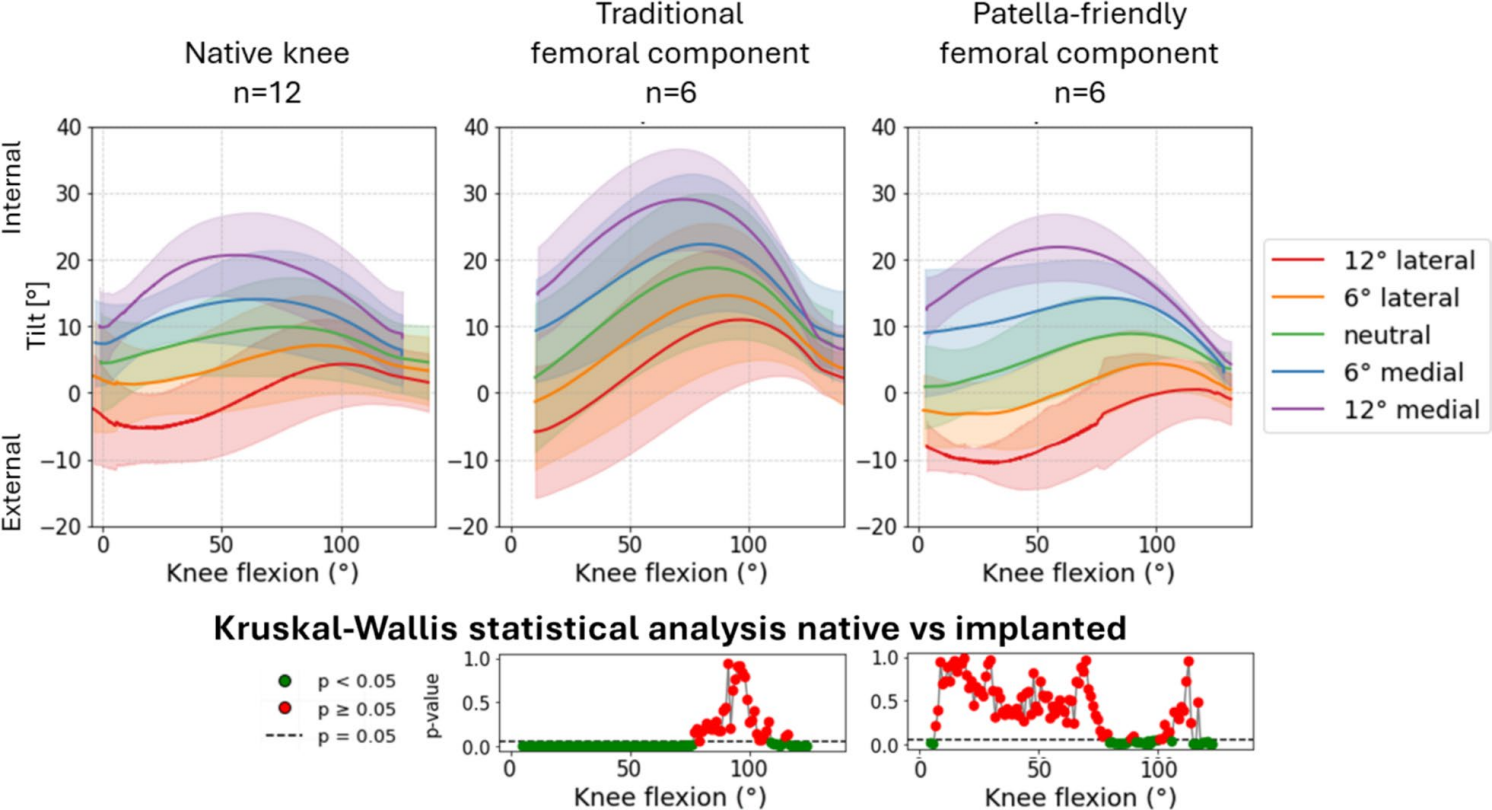
Internal–external tilt as a function of medial–lateral direction of the quadriceps force in native limbs (left), limbs implanted with traditional design TKA (center), and limbs implanted with patella-friendly design TKA (right). On the bottom the results of the mixed effect models statistical analysis for the presence of TKA versus native grouped for the traditional and patella-friendly design femoral component. The significant values are represented in green and the nonsignificant ones in red, and the threshold for significance (*p* = 0.05) is marked as a dashed line

Internal–external tilt of the patella (Fig. 13) was significantly affected by TKA mainly in the traditional implant group, with highly significant differences at all flexion angles (*p* < 0.001), except at full flexion where the effect was attenuated. Interactions between the presence of prosthesis and medial QV_ML_ (both 6° and 12°) showed high significance throughout most of the cycle, while lateral QV_ML_, only at flexed knee.

In the patella-friendly design group, the patellar tilt did not differed significantly from native (*p* > 0.05). Interactions with the direction of the quadriceps load were rarely significant.

**Fig. 14:**
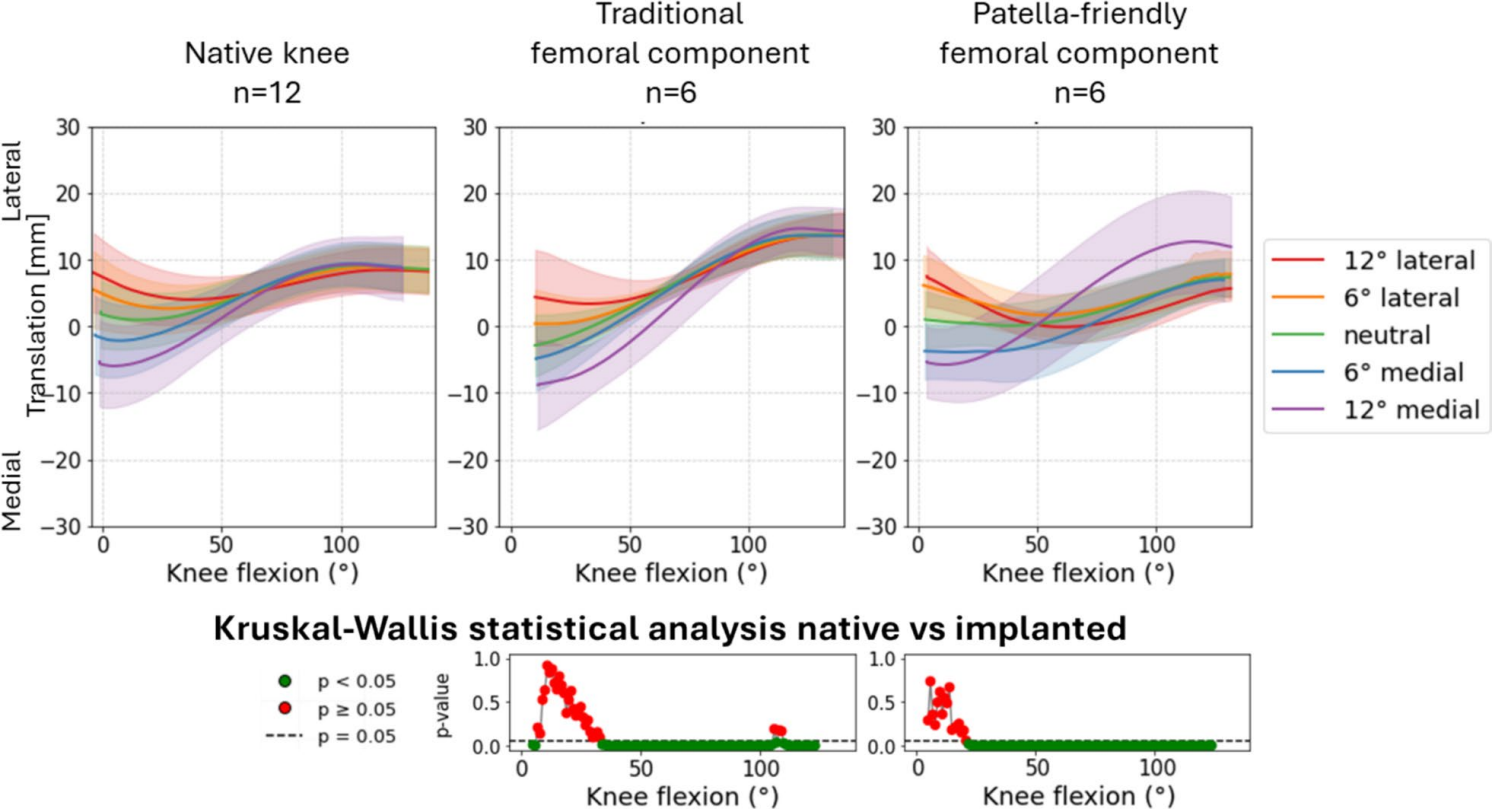
Variation of medial–lateral translation as a function of medial–lateral direction of the quadriceps force in native limbs (left), limbs implanted with traditional design TKA (center), and limbs implanted with patella-friendly design TKA (right). On the bottom the results of the mixed effect models statistical analysis for the presence of TKA versus native grouped for the traditional and patella-friendly design femoral component. The significant values are represented in green and the nonsignificant ones in red, and the threshold for significance (*p* = 0.05) is marked as a dashed line

For medial–lateral patellar translation (Fig. 14), the traditional design implant induced systematic deviations across flexion angles over 30° (*p* < 0.001), with significant interactions at the fully flexed knee for all directions and from mid-flexion for the neutral and medial directions.

The patella-friendly design component showed significant differences after the mid-flexion (30° to fully flexed, *p* < 0.05), and no significance at extended knee (*p* > 0.05). Interaction terms were generally not significant, indicating a more consistent response. The only exception was with a 12° medial QV_ML_ with a significant deviation (*p* < 0.001) from native kinematics.

## Discussions

This study investigated the influence of TKA on PF kinematics, with particular focus on how femoral component design and quadriceps loading affect patellar motion. Using an in vitro model with paired cadaveric knees, we compared native joints with those implanted with either a traditional design femoral component (Sphere, Medacta) or a newer patella-friendly design femoral component (SpheriKA, Medacta), under a range of physiologically and pathologically relevant loading conditions. Our findings provide valuable insight into how surgical choices and implant geometry affect postoperative PF biomechanics, and they help bridge the gap between clinical observations and biomechanical joint behavior.

### Influence of TKA on patellofemoral kinematics in the reference condition

First, we considered the reference condition, consisting of flexion–extension with minimal quadriceps load (20 N) and no deviations of the force vector direction. Under this condition, TKA did not significantly alter PF kinematics compared with the native knee. However, differences were observed in certain features, such as the range of motion in varus–valgus rotation between the two femoral component designs. Specimens implanted with the traditional design exhibited a broader range of patellar motion compared with the native condition, whereas those implanted with the patella-friendly design showed trajectories closer to the native pattern. This discrepancy may be explained by the different trochlear geometries: the traditional design, with its narrower and more constraining groove, guides patellar tracking throughout the entire flexion range but reproduces the native motion only when the prosthetic trochlea is aligned with the patient’s prearthritic joint line. With KA, this occurs only in subjects with a naturally neutral limb alignment; in knees with constitutional varus or valgus, the patella is forced to follow a nonphysiological path [9]. In contrast, the patella-friendly design, with its wider, funnel-shaped trochlea, allows the patella more freedom in full extension, while gradually constraining and guiding it during flexion. This progressive engagement, allowed by the presence of an elevated lateral flange accommodates variations in individual anatomy, reducing the risk of nonphysiological tracking in knees with constitutional varus or valgus.

The development of patella-friendly KA-specific designs addresses patients with atypical anatomy, for whom standard alignment may not yield optimal outcomes [9]. Clinical studies report similar functional results between MA and KA in patients with neutral native alignment, such as that reproduced in our reference condition. In contrast, patients with native varus or valgus alignment show divergent outcomes depending on the alignment strategy adopted [23]. For this reason, in subsequent sessions we evaluated the response of patellar tracking to QV variations in load and directions.

Despite variability in individual patterns, several common features were observed. Native knees consistently displayed an early medial shift during initial flexion, which was absent in both implant types. Implanted knees instead showed a progressive lateral translation from the start of flexion. Even if this could be partially related to the absence of hyperextension in the knees after TKA implantation, our results are consistent with earlier in vitro studies [24, 25] that also found a reduced medial shift in unresurfaced patella TKA with an earlier lateral translation. Previous studies also reported changes in the internal–external tilt [25, 26], that were not observed to be significant with these implant designs.

### Role of quadriceps loading direction and magnitude

Given the patient-specific nature of the quadriceps vector, an important aspect of our analysis involved assessing the role of quadriceps loading direction and magnitude in all the implanted specimens. Quadriceps direction had the strongest effect on PF kinematics in both implant groups. QV_ML_ influenced varus–valgus rotation, tilt, and medial–lateral translation across most of the flexion cycle. QV_AP_ and load magnitude had more localized effects, primarily in early/mid-flexion and on vertical patellar position. This general behavior mirrors previous findings in native knees [16].

Interestingly, a reversal in the varus–valgus trend was observed around 60° of flexion for all the specimens, marking a point of patellar stabilization within the femoral groove. This finding aligns with our previous observations in native knees [16]. In the previous work, the 50–60° flexion range was associated with a biomechanical equilibrium point in the native knee, where increased trochlear congruency provides greater constraint, allowing the patella to shift trajectory in response to QV direction.

Generally, our findings emphasize that QV_ML_ is a dominant determinant of patellar behavior, underscoring the clinical relevance of restoring the native Q angle, which varies substantially among patients.

### Design-specific behavior under variable QV direction

Differences between implant designs became more evident under non-neutral loading. Traditional components exhibited a larger rotational range in varus–valgus and tilt—particularly under medially directed quadriceps loading—compared with both the native knee and the patella-friendly design. A wider patellar rotation in traditional implants may contribute to anterior knee pain and PF syndromes, both in case of varus–valgus rotation [27] and tilt [28].

Furthermore, the higher interaction between the presence of TKA and the action of the quadriceps muscle in case of traditional femoral component design may reflect reduced tolerance to variations in Q angle. These findings suggest that traditional design components, which are typically optimized for mechanical alignment and feature a standardized 6° trochlear angle, may not accommodate the restored Q angle achieved through the kinematic alignment. This mismatch likely contributes to altered PF tracking and may help explain the persistent clinical reports of anterior knee pain and crepitus associated with some TKA implants [2].

The patella-friendly design also showed limitations, in particular under extreme medial QV_ML_ (12°). Half of the specimens implanted with the patella-friendly design component could not be tested at 12° medial direction owing to complications in extensor mechanism behavior, suggesting a design limitation with extreme loading scenarios. In this loading condition, even successful trials showed abnormal medial–lateral motion. In several cases, the patella failed to engage properly with the trochlear groove, translating along the lateral wall of the trochlea. Proper trochlear engagement is essential for patellar stability and efficient load transfer through the extensor mechanism [27]. As all failures occurred in the patella-friendly group, we also repeated the mixed-effects analysis after excluding all trials from the donors in which failure was observed, not only the failed trials. This sensitivity analysis, performed on 90 trials from the three remaining specimens, confirmed that the overall pattern of significant differences between native and implanted conditions did not change: the flexion-angle intervals showing statistical significance remained substantially the same. This supports the robustness of our findings despite the missing data at extreme medial loading.

Traditional design implants, in contrast, displayed larger and more frequent deviations across QV angles, especially in varus–valgus and tilt. However, they showed better mechanical containment with extreme medial QV, reducing the risk of excessive soft tissue strain. These findings suggest that while anatomical trochlear designs improve tolerance to patient-specific Q angles, they may still be challenged in extreme varus morphotypes.

### Key findings and clinical implications

These findings carry direct implications for patient-specific implant selection, strongly supporting the hypothesis that patella-friendly designs are better suited for patients with larger Q angles or valgus morphotypes. The specific geometry of the patella-friendly component (featuring a wider, funnel-shaped trochlea and an elevated lateral flange) proves particularly advantageous in these cases. This design effectively accommodates a lateralized quadriceps vector, guiding the patella into engagement without overconstraint and thereby more effectively restoring native-like kinematics [13]. Conversely, the traditional design, while demonstrating superior containment under extreme medial loading scenarios, as in severe varus morphotypes, achieved this stability at the cost of significant deviations from native patellar motion.

This tradeoff underscores a critical consideration for surgeons. The patella-friendly design, optimized for kinematic alignment (KA), appears ideal for most patients undergoing KA-TKA. However, its relative lack of medial containment, which explains the instability observed under extreme medial load, suggests that it may pose a risk for individuals with extreme native varus alignment. These patients are rarely candidates for unrestricted KA: a shift towards a restricted kinematic or even mechanical alignment strategy is more common, with a standard femoral component offering more inherent medial constraint [29]. Therefore, implant choice should be guided by a preoperative assessment of the patient’s native alignment and expected quadriceps vector, moving towards a truly personalized arthroplasty strategy.

Several clinical long-term studies [11, 12, 14, 15] have shown a small reduction of patella-related complications, such as crepitus and anterior knee pain, associated with patella-friendly designs compared with traditional components. However, most of these data are epidemiological or observational and lack direct biomechanical substantiation. Our experimental results demonstrate that patella-friendly femoral components better preserve native-like varus–valgus alignment and medial–lateral translation under neutral load, affirming the clinical hypotheses.

### Limitations

This study presents several limitations that should be acknowledged. First, the in vitro setup does not replicate dynamic muscle cocontraction or neuromuscular control, and the magnitude of the applied loads remains substantially lower than in vivo conditions. The quadriceps was modeled as a single resultant force vector, a simplification commonly used in literature [30–37]. Even if this approach prevents distinguishing the specific contributions of individual quadriceps components—particularly the vastus medialis and lateralis—to patellar stability, it reduces experimental uncertainties, while having more control over the applied parameters.

A further limitation is the sample size, which—although comparable to other biomechanical cadaveric studies—remains relatively small and may contribute to the interspecimen variability observed in the results. Furthermore, the use of contralateral specimens allows for more direct comparison of the effect of different implant designs, under the assumption, partially confirmed with the native condition tests, that contralateral joints might behave similarly.

Another critical aspect concerns measurement uncertainty. The registration pipeline relied on multiple CT-based registrations combined with optoelectronic tracking. Although each registration was validated by minimizing point-to-point distances and ensuring submillimetric residuals, estimating the overall cumulative error remains challenging.

Another key point is that we did not resurface the patella, a variable that may affect clinical outcomes and patellar contact behavior. Future research should extend this analysis by also evaluating the behavior of the PF joint after patellar resurfacing. In addition, an assessment of contact forces [38] as well as motion could be important to improve prosthetic designs, and thus the quality of life of patients with TKA.

## Conclusions

This in vitro study demonstrates that the choice of femoral component design in KA-TKA is critical for restoring physiological PF biomechanics. The key practical takeaway is that a “patella-friendly” design, with its wider and more anatomically oriented trochlea, generally provides superior replication of native patellar kinematics and greater robustness to variations in quadriceps loading direction compared with a traditional design. This supports its use as the preferred component for most KA-TKA procedures, particularly in patients with valgus alignment or a laterally directed quadriceps force.

However, a crucial caveat was identified: this design may be susceptible to instability under extreme medial loading conditions, suggesting a potential risk in patients with severe varus morphology. Therefore, a patient-specific approach is paramount, guided by preoperative assessment of limb alignment and extensor mechanism dynamics.

## Supplementary Information

Supplementary material 1.

Supplementary material 2.

Supplementary material 3.

Supplementary material 4.

## Data Availability

The data that support the findings of this study are available upon reasonable request.

## Abbreviations

PF: Patellofemoral
TKA: Total knee arthroplasty
MA: Mechanical alignment
KA: Kinematic alignment
QV: Quadriceps vector
QV_ML_: Medial–lateral direction of the quadriceps vector
QV_AP_: Anterior–posterior direction of the quadriceps vector
QV_load_: Force magnitude of the quadriceps vector
