# Supplementary material for: Effect of femoral component design and quadriceps load on patellofemoral kinematics after total knee arthroplasty: an in vitro cadaveric study
Source: Knee Surg Relat Res. 2026 Feb 26;38:9. doi: 10.1186/s43019-026-00308-6 (PMC12937521; doi:10.1186/s43019-026-00308-6)
Supplement: Supplementary file 1 — Supplementary material 1. [file 43019_2026_308_MOESM1_ESM.docx]

# SUPPLEMENTARY MATERIALS

Appendix to the paper:

Effect of Femoral Component Design and Quadriceps Load on Patellofemoral Kinematics After Total Knee Arthroplasty: An In Vitro Cadaveric Study

## **SUPPLEMENTARY MATERIAL #1**

### **Influence of the quadriceps muscle for all degrees of freedom of the patellofemoral joint after TKA**

This section provides a comprehensive visualization of all six kinematic components (Figure S1_1) of the patellofemoral joint following TKA, expanding upon the results presented in the main manuscript. Specifically, the plots show the median ± standard deviation across all specimens, grouped according to the three investigated quadriceps control parameters: QV_ML_ (line of action direction in the frontal plane), QV_AP_ (line of action direction in the sagittal plane), and QV_load_ (muscle force magnitude).
Additionally, the variation of each kinematic parameter relative to the reference test (neutral direction and 20N load) is plotted, again as median ± SD across specimens.
Finally, statistical significance of the effect of each quadriceps parameter on patellofemoral motion is assessed using the Kruskal-Wallis test with Bonferroni correction, and corresponding p-values are reported for all degrees of freedom (3 translations + 3 rotations).


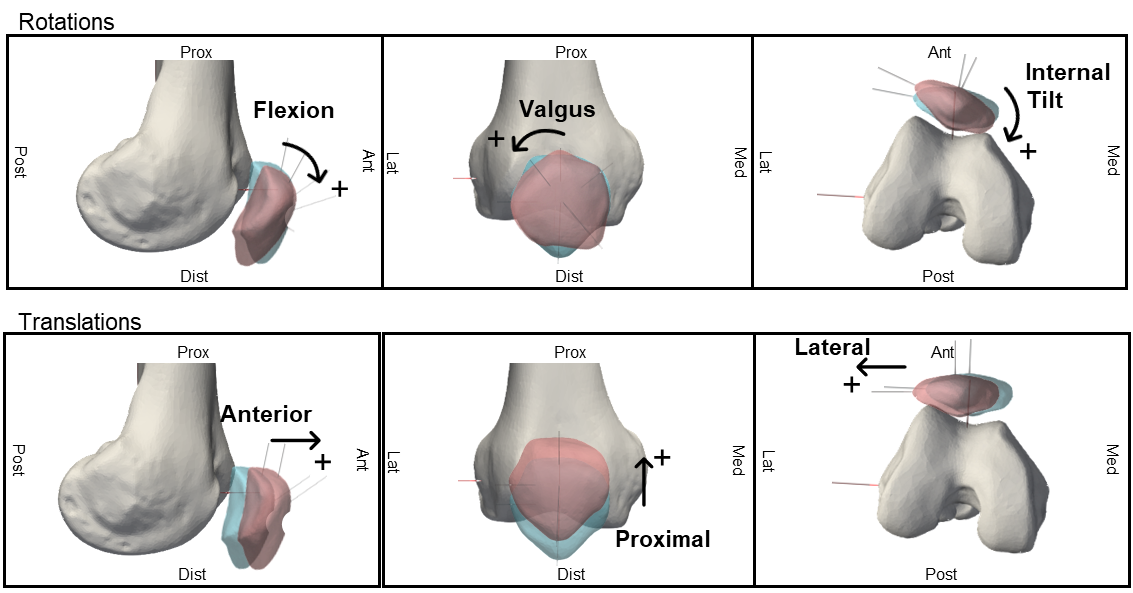


Figure S1_1: Motion components of the patellofemoral joint of a right leg. Figshare repository (10.6084/m9.figshare.30019969).


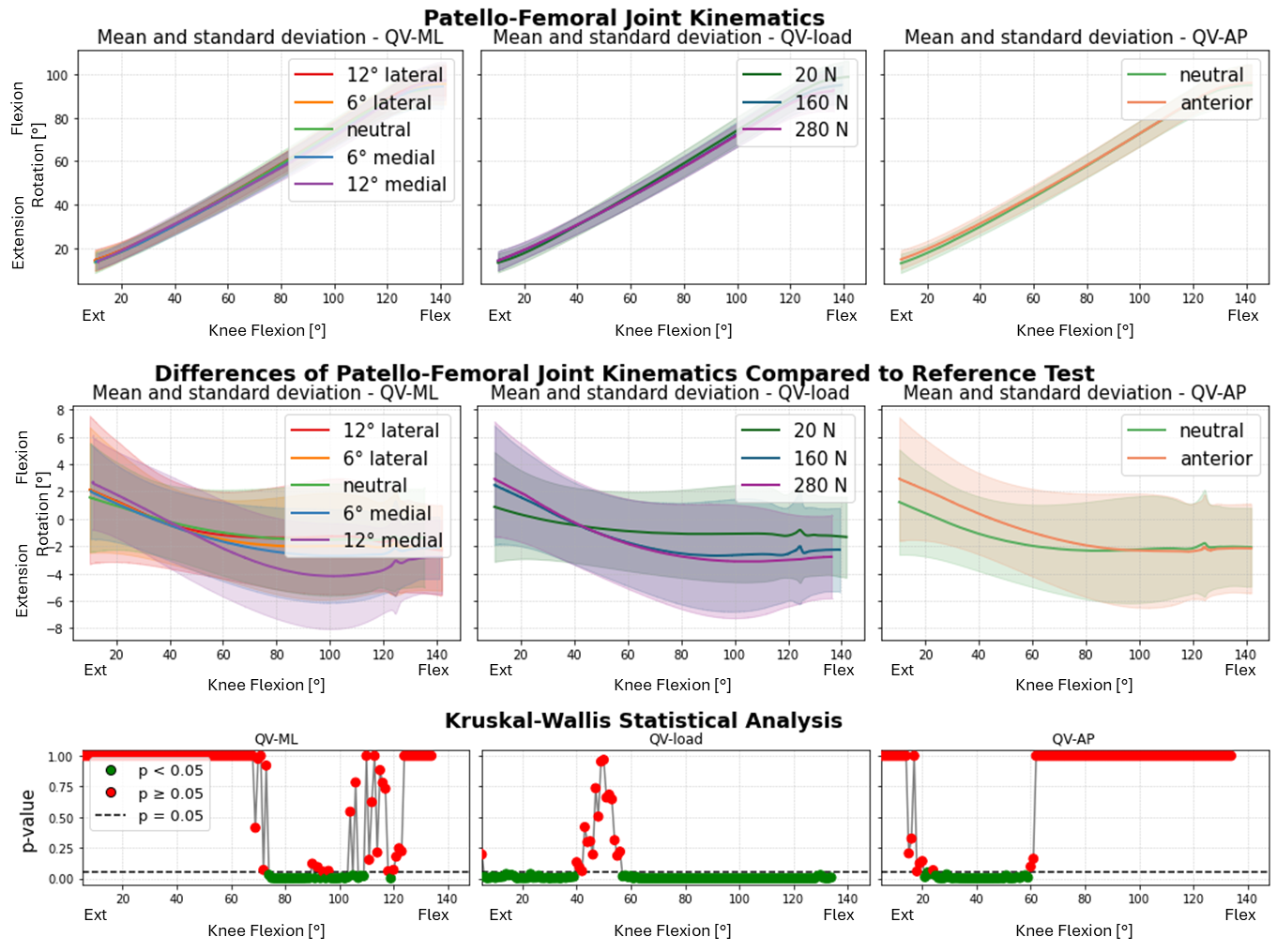


Figure S1_2: Flexion-Extension rotation of the patella after TKA without patellar resurfacing. TOP: absolute values as a function of knee flexion angle (the median and standard deviation between 12 specimens are plotted). CENTER: differences of all tests compared with the reference test (QVload = 20 N, QVML = neutral, QVAP = posterior). Left shows the difference as a function of QVML, middle QVload, right QVAP. BOTTOM: significance of the differences plotted at the center. The p-value trend is plotted for the three parameters (left QVML, middle QVload, right QVAP); the significant values are highlighted in green (p<0.05), the non significant ones in red (p≥0.05).


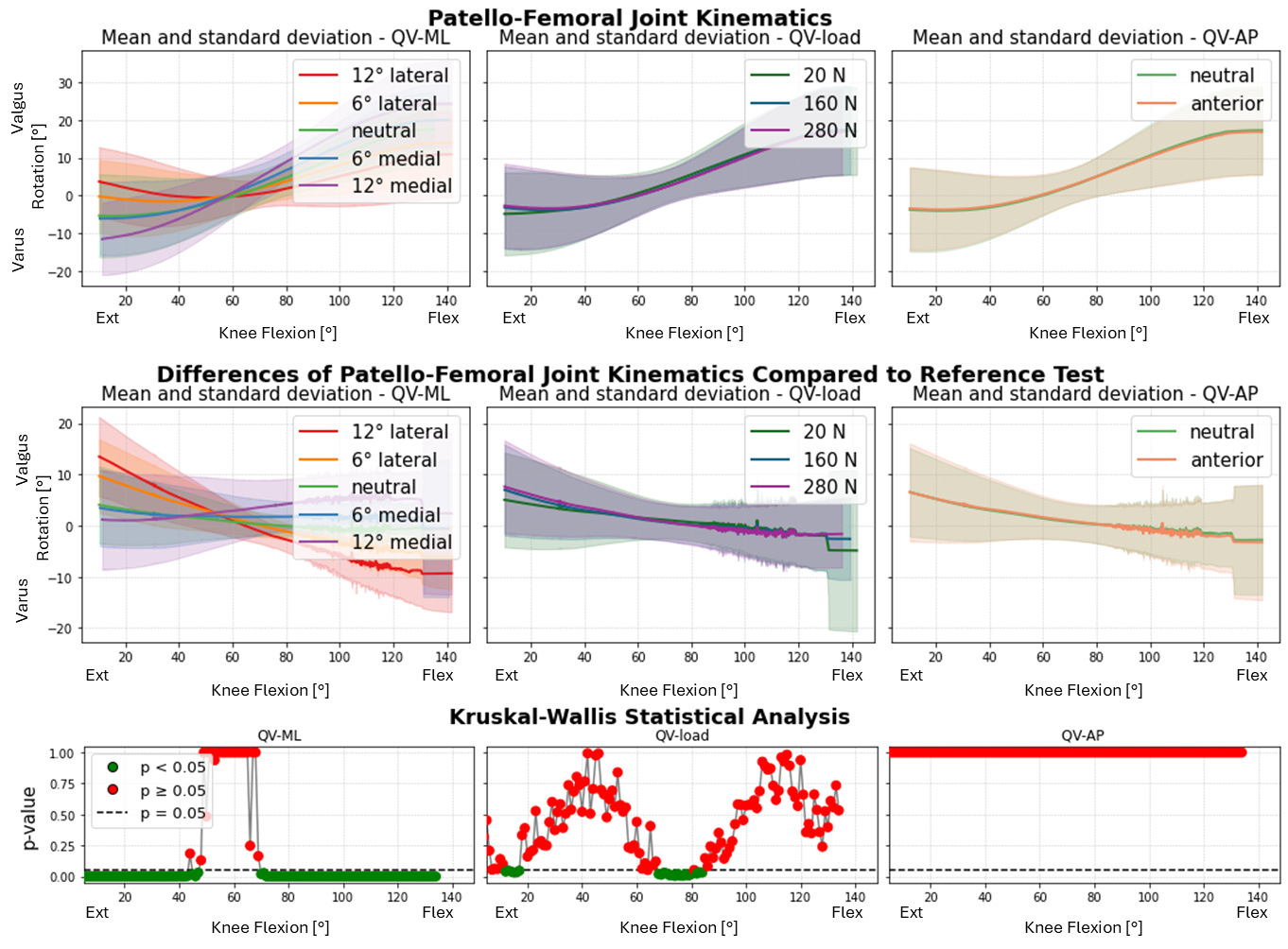


Figure S1_3: Varus-valgus rotation of the patella after TKA without patellar resurfacing. TOP: absolute values as a function of knee flexion angle (the median and standard deviation between 12 specimens are plotted). CENTER: differences of all tests compared with the reference test (QVload = 20 N, QVML = neutral, QVAP = posterior). Left shows the difference as a function of QVML, middle QVload, right QVAP. BOTTOM: significance of the differences plotted at the center. The p-value trend is plotted for the three parameters (left QVML, middle QVload, right QVAP); the significant values are highlighted in green (p<0.05), the non significant ones in red (p≥0.05).


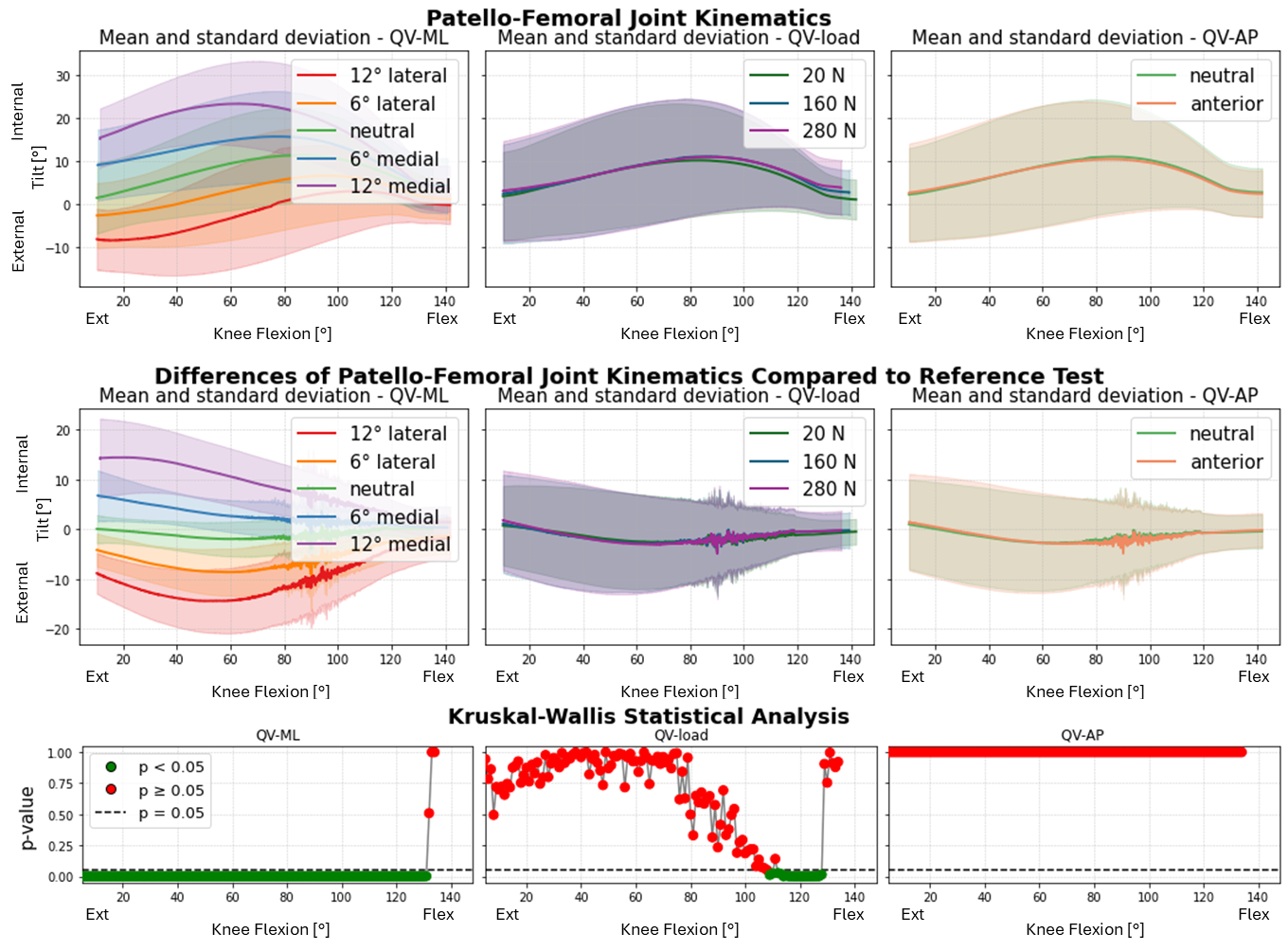


Figure S1_4: Internal-External of the patella after TKA without patellar resurfacing. TOP: absolute values as a function of knee flexion angle (the median and standard deviation between 12 specimens are plotted). CENTER: differences of all tests compared with the reference test (QVload = 20 N, QVML = neutral, QVAP = posterior). Left shows the difference as a function of QVML, middle QVload, right QVAP. BOTTOM: significance of the differences plotted at the center. The p-value trend is plotted for the three parameters (left QVML, middle QVload, right QVAP); the significant values are highlighted in green (p<0.05), the non significant ones in red (p≥0.05).


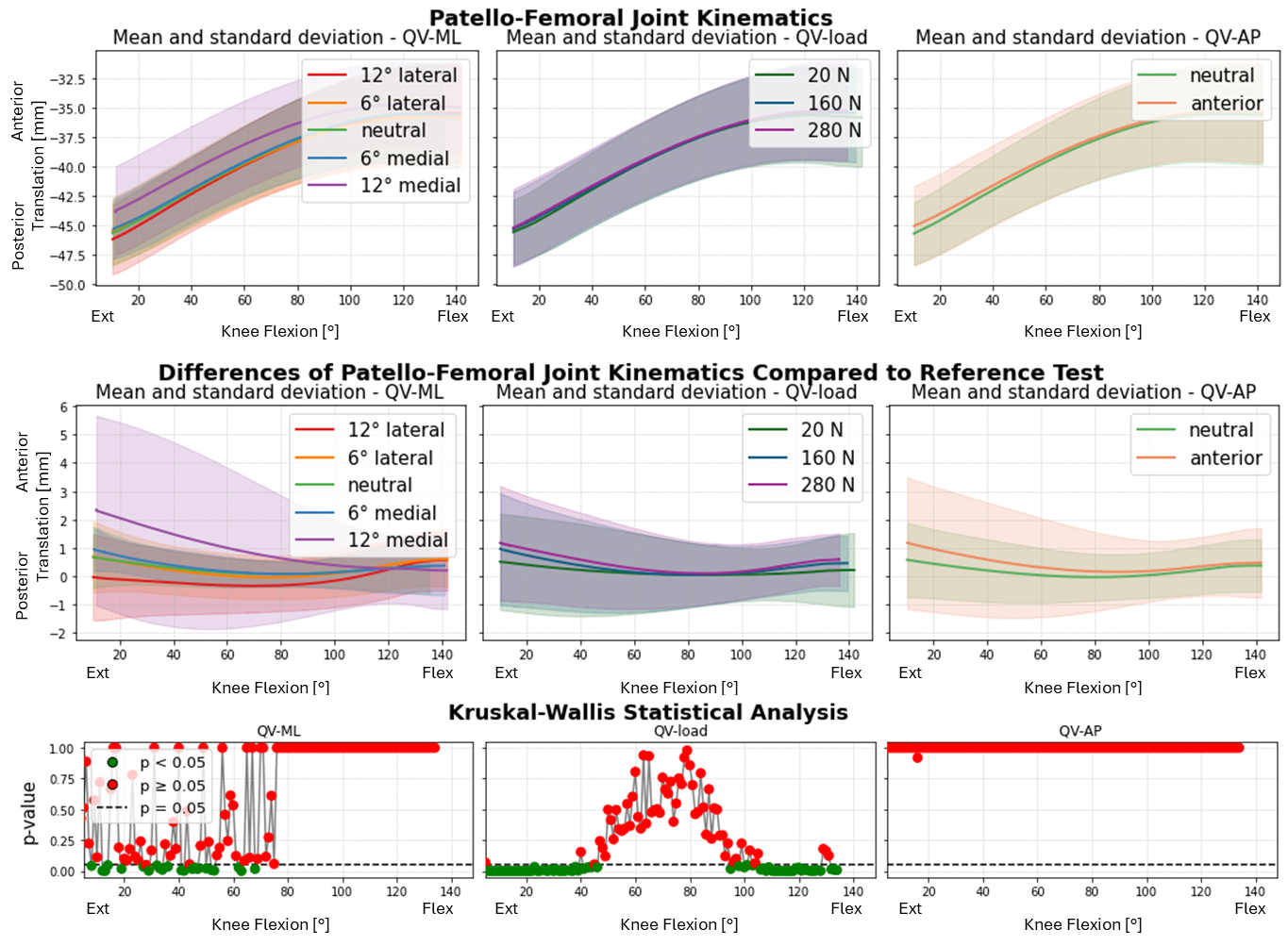


Figure S1_5: Anterior-Posterior translation of the patella after TKA without patellar resurfacing. TOP: absolute values as a function of knee flexion angle (the median and standard deviation between 12 specimens are plotted). CENTER: differences of all tests compared with the reference test (QVload = 20 N, QVML = neutral, QVAP = posterior). Left shows the difference as a function of QVML, middle QVload, right QVAP. BOTTOM: significance of the differences plotted at the center. The p-value trend is plotted for the three parameters (left QVML, middle QVload, right QVAP); the significant values are highlighted in green (p<0.05), the non significant ones in red (p≥0.05).


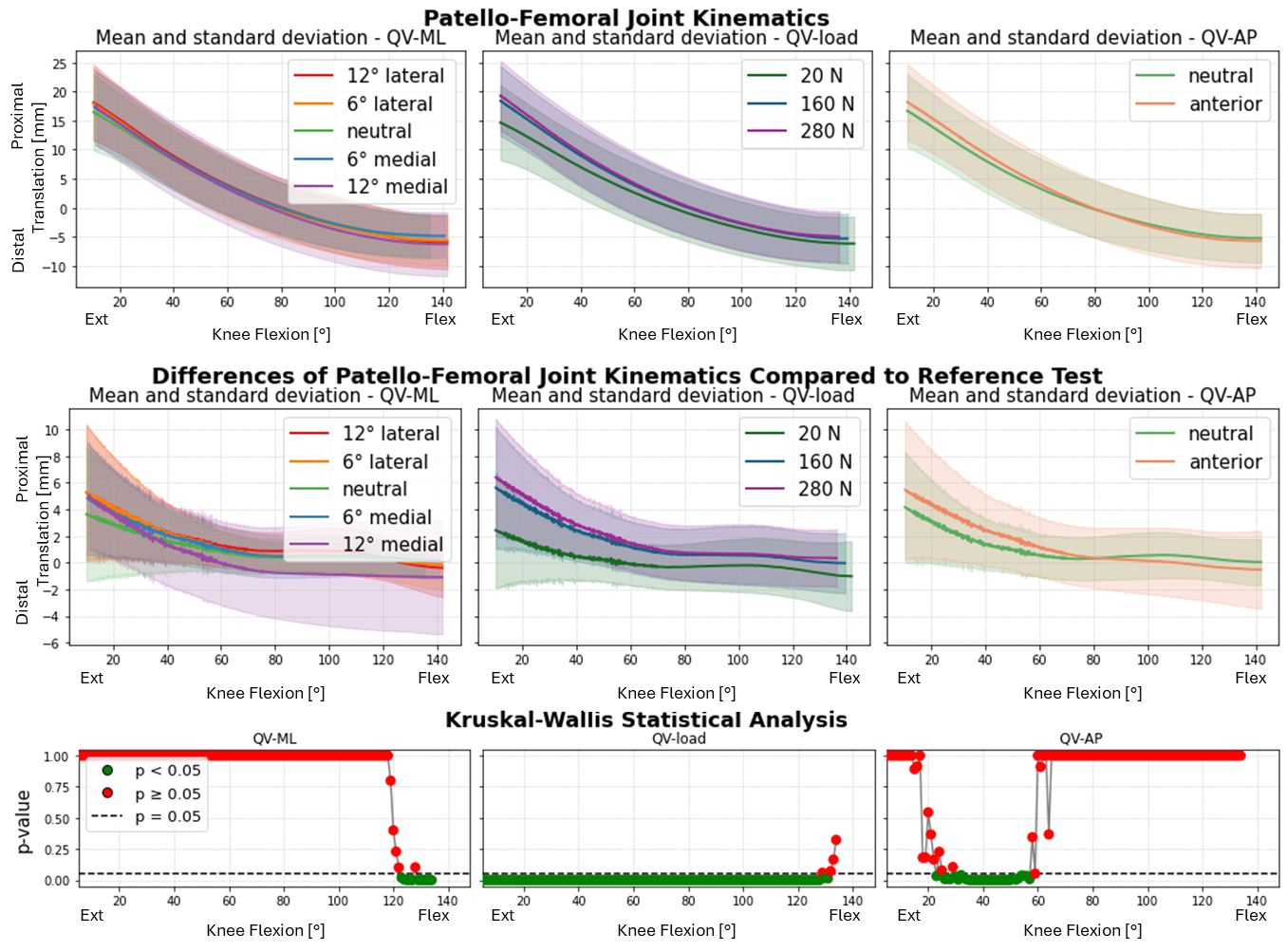


Figure S1_6: Proximal-Distal translation of the patella after TKA without patellar resurfacing. TOP: absolute values as a function of knee flexion angle (the median and standard deviation between 12 specimens are plotted). CENTER: differences of all tests compared with the reference test (QVload = 20 N, QVML = neutral, QVAP = posterior). Left shows the difference as a function of QVML, middle QVload, right QVAP. BOTTOM: significance of the differences plotted at the center. The p-value trend is plotted for the three parameters (left QVML, middle QVload, right QVAP); the significant values are highlighted in green (p<0.05), the non significant ones in red (p≥0.05).


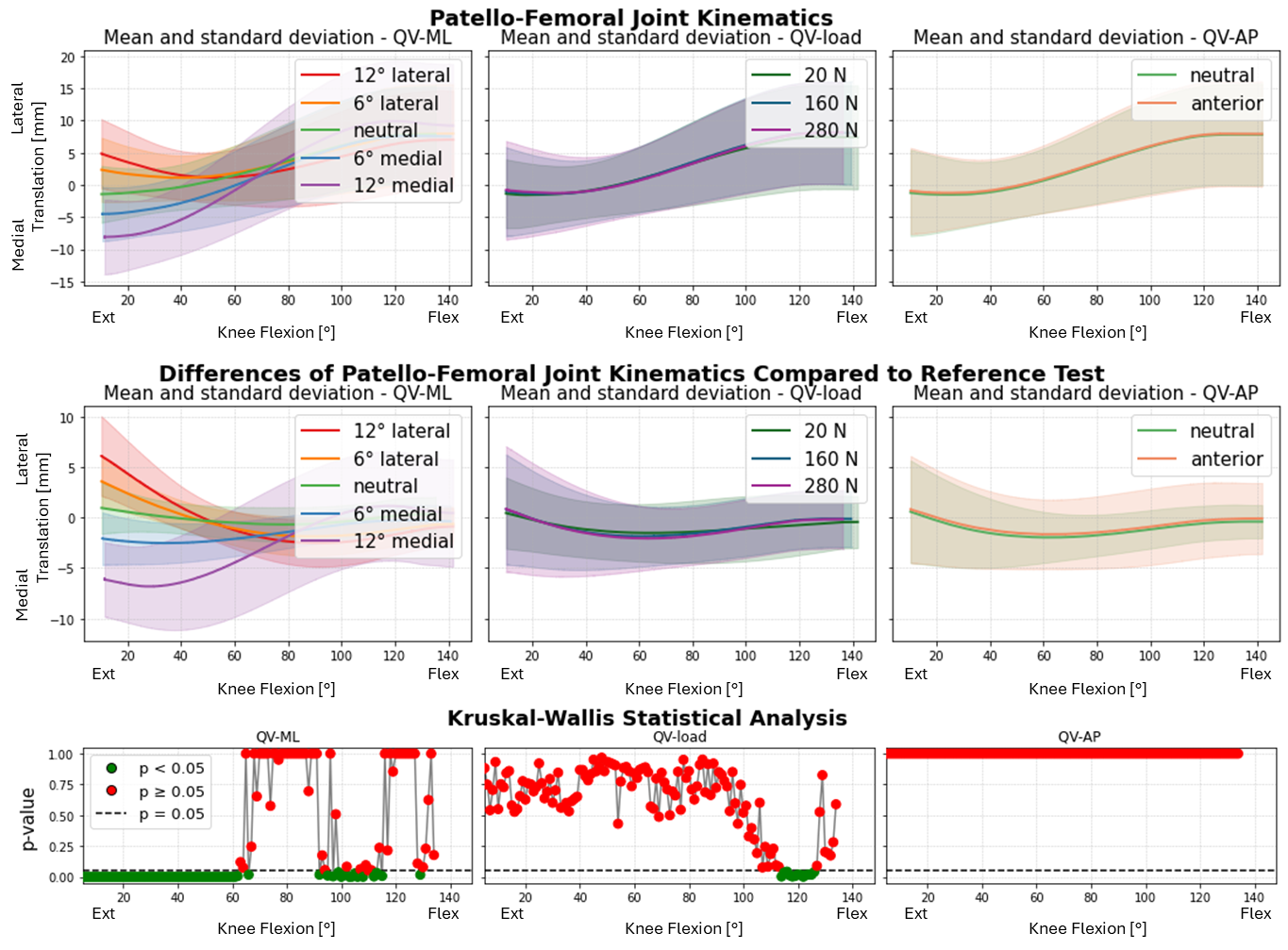


Figure S1_7: Medial-Lateral translation of the patella after TKA without patellar resurfacing. TOP: absolute values as a function of knee flexion angle (the median and standard deviation between 12 specimens are plotted). CENTER: differences of all tests compared with the reference test (QVload = 20 N, QVML = neutral, QVAP = posterior). Left shows the difference as a function of QVML, middle QVload, right QVAP. BOTTOM: significance of the differences plotted at the center. The p-value trend is plotted for the three parameters (left QVML, middle QVload, right QVAP); the significant values are highlighted in green (p<0.05), the non significant ones in red (p≥0.05).
