# Supplementary material for: Effect of femoral component design and quadriceps load on patellofemoral kinematics after total knee arthroplasty: an in vitro cadaveric study
Source: Knee Surg Relat Res. 2026 Feb 26;38:9. doi: 10.1186/s43019-026-00308-6 (PMC12937521; doi:10.1186/s43019-026-00308-6)
Supplement: Supplementary file 2 — Supplementary material 2. [file 43019_2026_308_MOESM2_ESM.docx]

# SUPPLEMENTARY MATERIALS

Appendix to the paper:

Effect of Femoral Component Design and Quadriceps Load on Patellofemoral Kinematics After Total Knee Arthroplasty: An In Vitro Cadaveric Study

## **SUPPLEMENTARY MATERIAL #2**

### **Influence of the quadriceps muscle for all degrees of freedom of the tibiofemoral joint after TKA**

This section mirrors the analysis presented for the patellofemoral joint but focuses on the tibiofemoral articulation following TKA. The same graphical structure is adopted, reporting:

- Median ± SD of all six tibiofemoral kinematic parameters across the flexion-extension cycle, grouped by QVML, QVAP, and QVload.
- Median ± SD of the variation relative to the baseline condition.
- P-values from the Kruskal-Wallis test with Bonferroni correction for each parameter, identifying which quadriceps factors significantly influenced tibiofemoral motion.


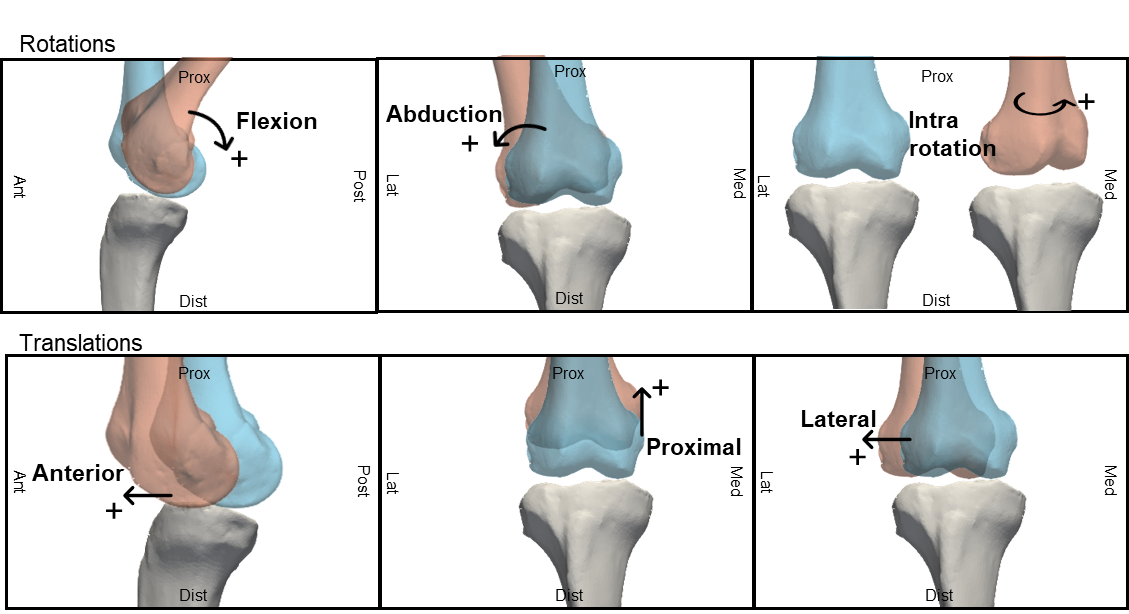
 Figure S2_1 Degrees of freedom of the tibiofemoral joint. Figshare repository (https://doi.org/10.6084/m9.figshare.29278721).


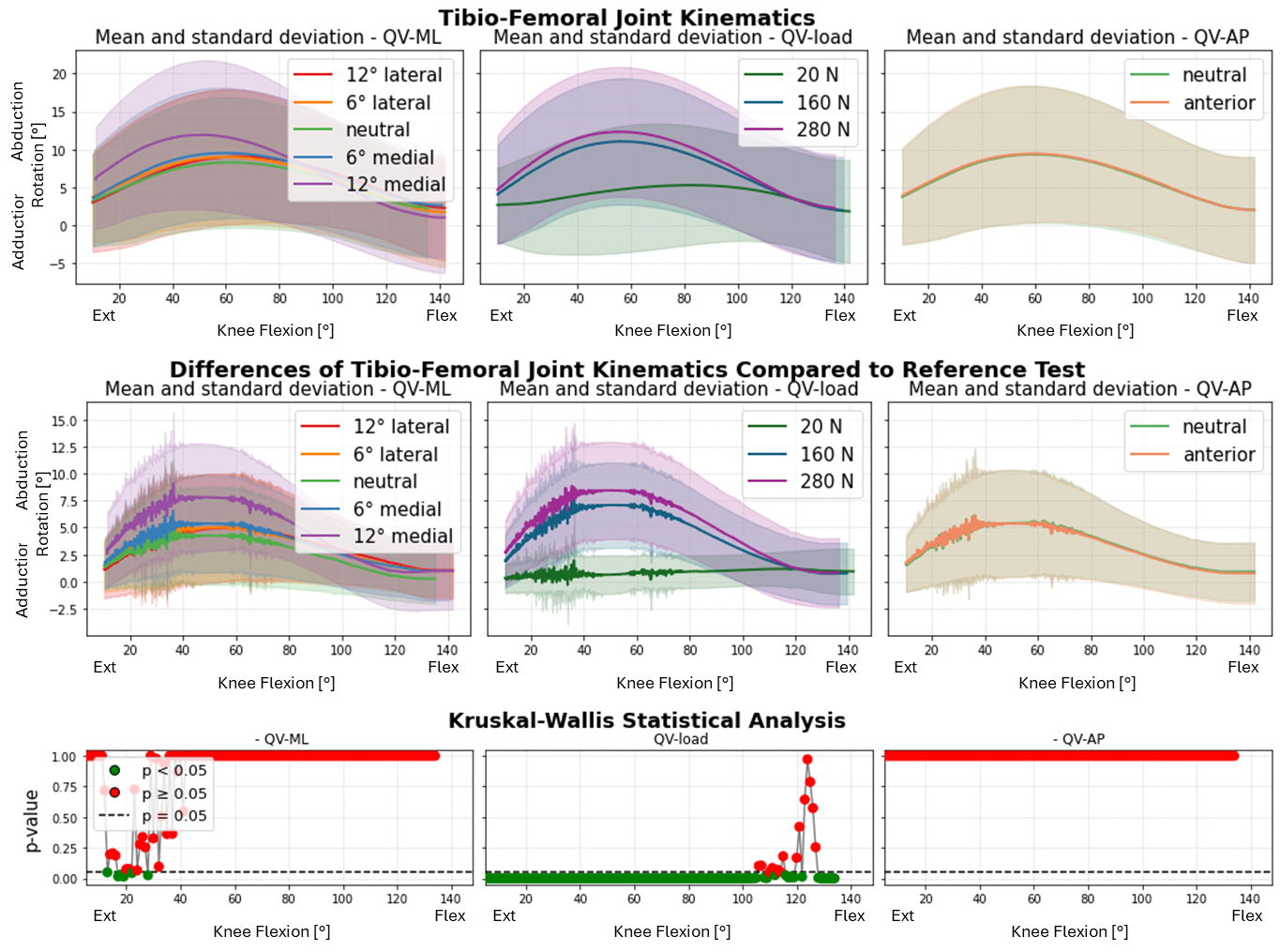


Figure S2_2: Abduction-Adduction rotation of the femur after TKA. TOP: absolute values as a function of knee flexion angle (the median and standard deviation between 12 specimens are plotted). CENTER: differences of all tests compared with the reference test (QVload = 20 N, QVML = neutral, QVAP = posterior). Left shows the difference as a function of QVML, middle QVload, right QVAP. BOTTOM: significance of the differences plotted at the center. The p-value trend is plotted for the three parameters (left QVML, middle QVload, right QVAP); the significant values are highlighted in green (p<0.05), the non significant ones in red (p≥0.05).


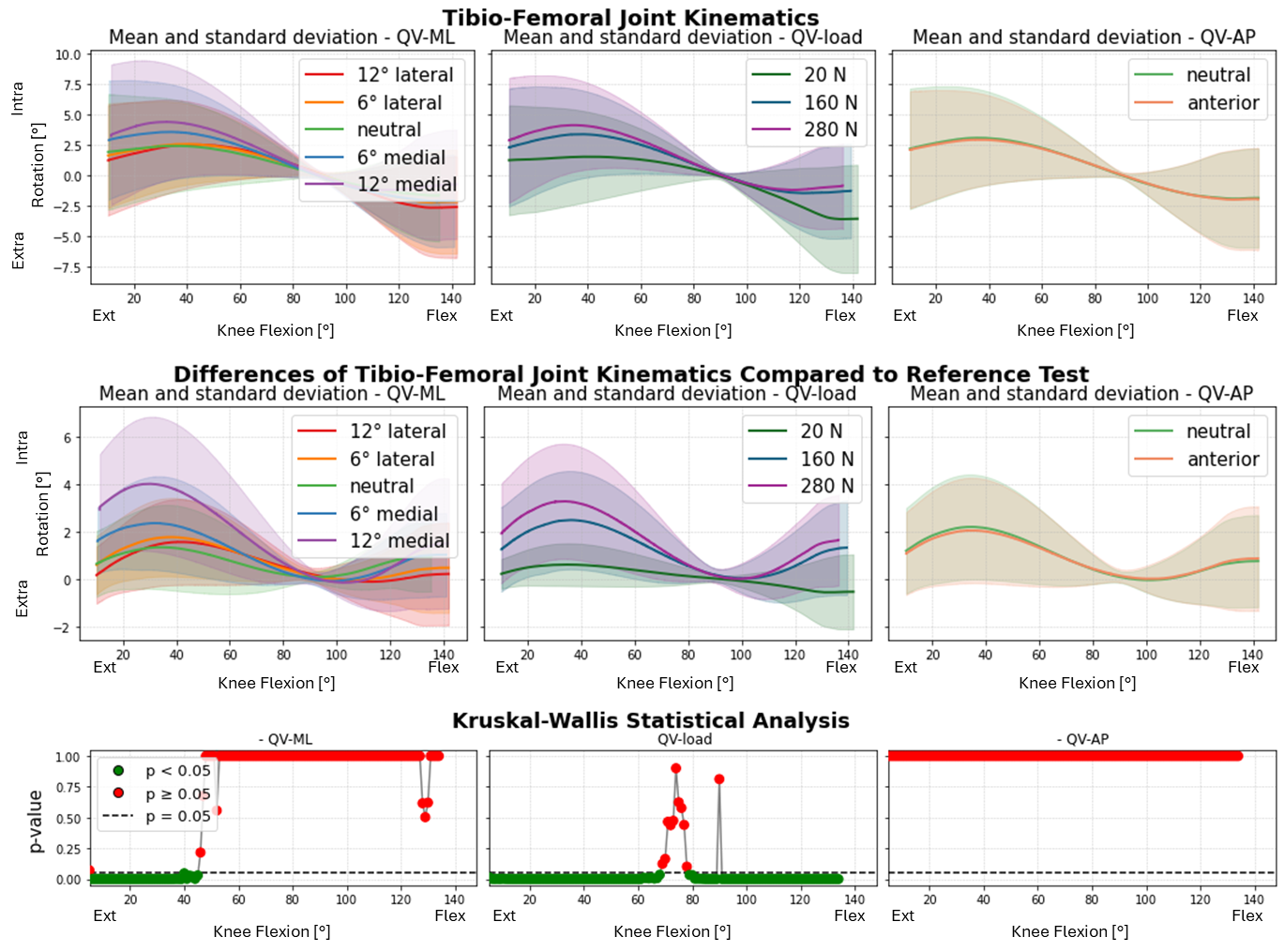


Figure S2_3: Intra-Extra rotation of the femur after TKA. TOP: absolute values as a function of knee flexion angle (the median and standard deviation between 12 specimens are plotted). CENTER: differences of all tests compared with the reference test (QVload = 20 N, QVML = neutral, QVAP = posterior). Left shows the difference as a function of QVML, middle QVload, right QVAP. BOTTOM: significance of the differences plotted at the center. The p-value trend is plotted for the three parameters (left QVML, middle QVload, right QVAP); the significant values are highlighted in green (p<0.05), the non significant ones in red (p≥0.05).


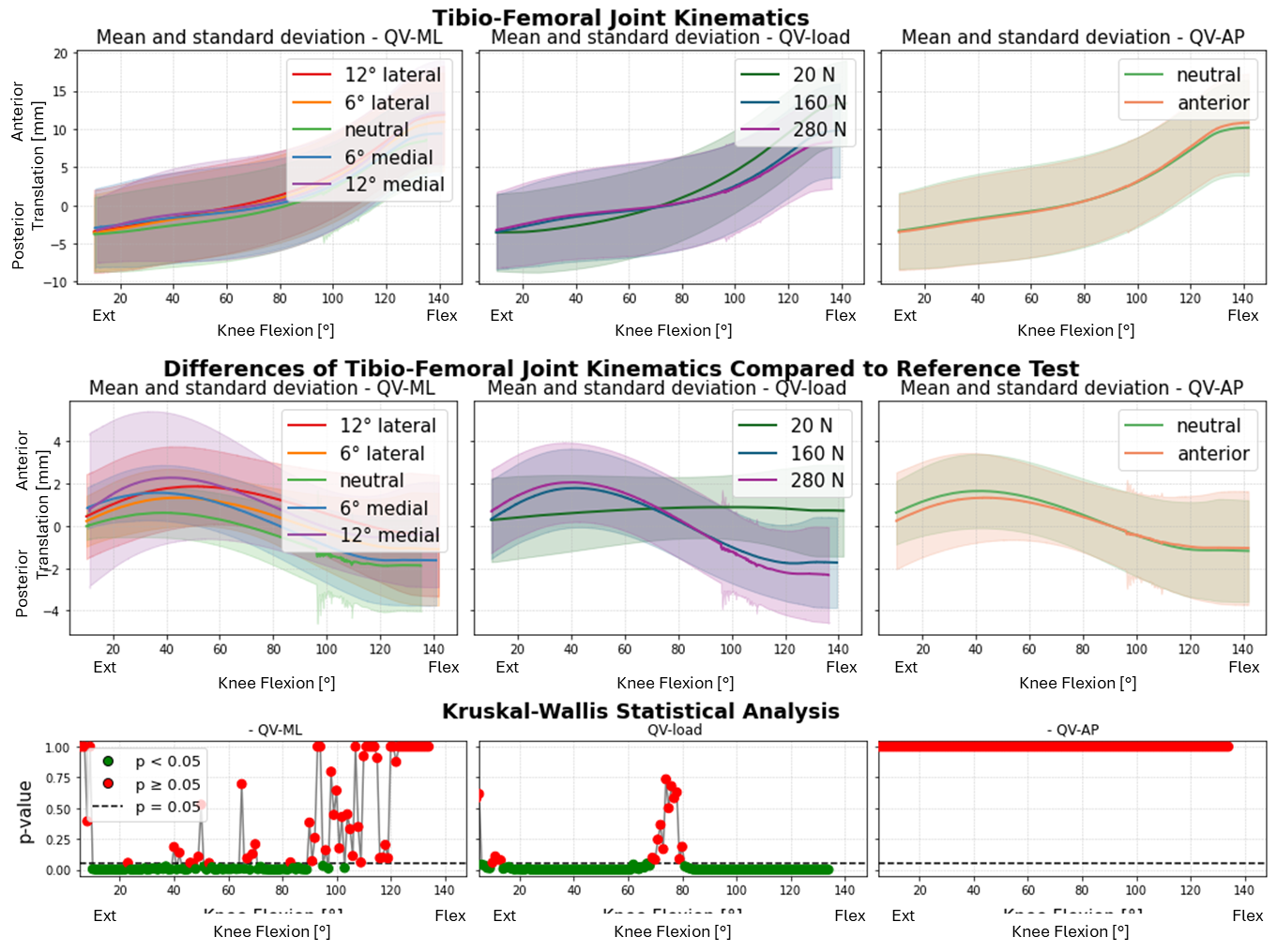


Figure S2_4: Anterior-Posterior translation of the femur after TKA. TOP: absolute values as a function of knee flexion angle (the median and standard deviation between 12 specimens are plotted). CENTER: differences of all tests compared with the reference test (QVload = 20 N, QVML = neutral, QVAP = posterior). Left shows the difference as a function of QVML, middle QVload, right QVAP. BOTTOM: significance of the differences plotted at the center. The p-value trend is plotted for the three parameters (left QVML, middle QVload, right QVAP); the significant values are highlighted in green (p<0.05), the non significant ones in red (p≥0.05).


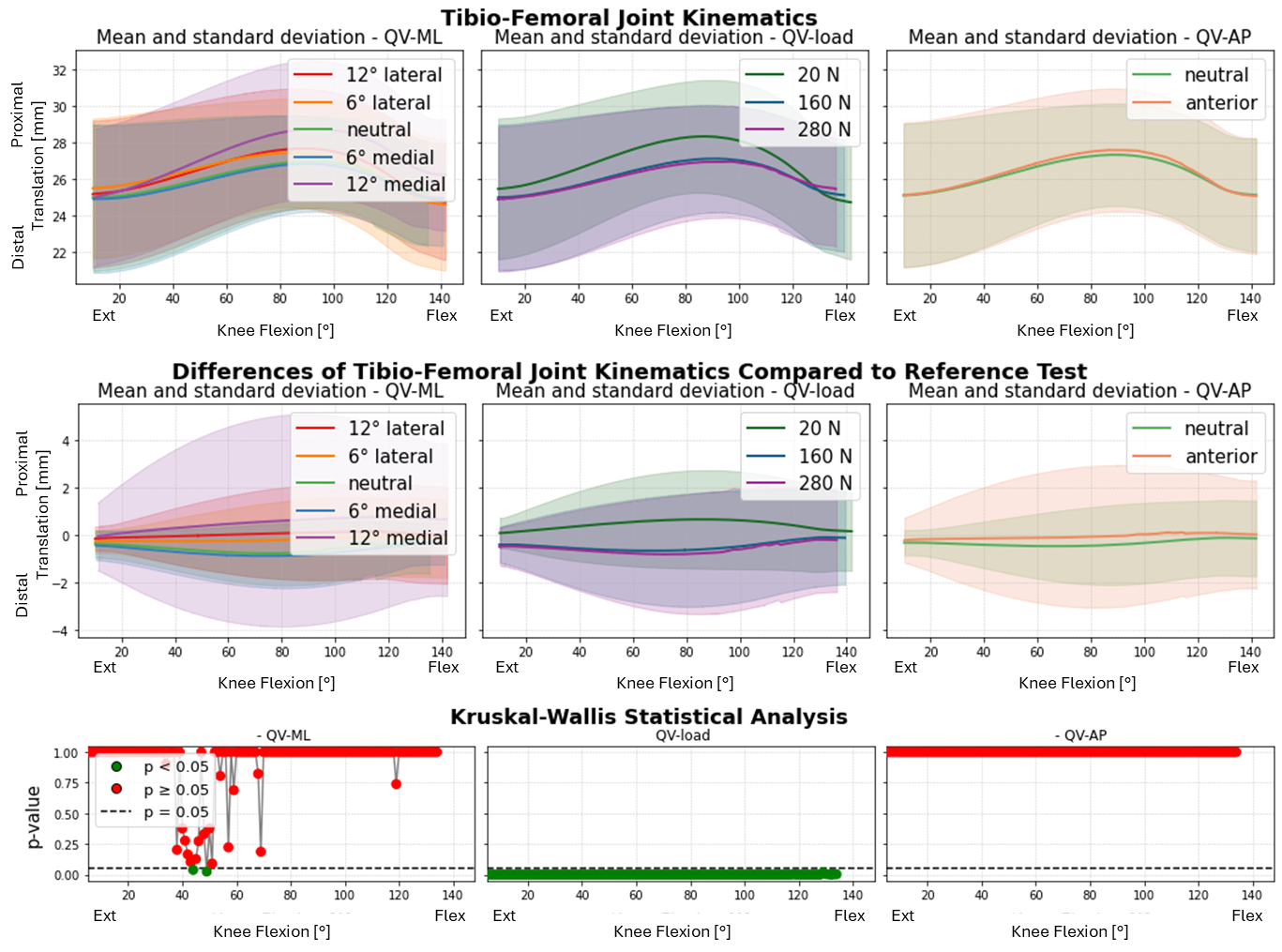


Figure S2_5: Proximal-Distal translation of the femur after TKA. TOP: absolute values as a function of knee flexion angle (the median and standard deviation between 12 specimens are plotted). CENTER: differences of all tests compared with the reference test (QVload = 20 N, QVML = neutral, QVAP = posterior). Left shows the difference as a function of QVML, middle QVload, right QVAP. BOTTOM: significance of the differences plotted at the center. The p-value trend is plotted for the three parameters (left QVML, middle QVload, right QVAP); the significant values are highlighted in green (p<0.05), the non significant ones in red (p≥0.05).green line.


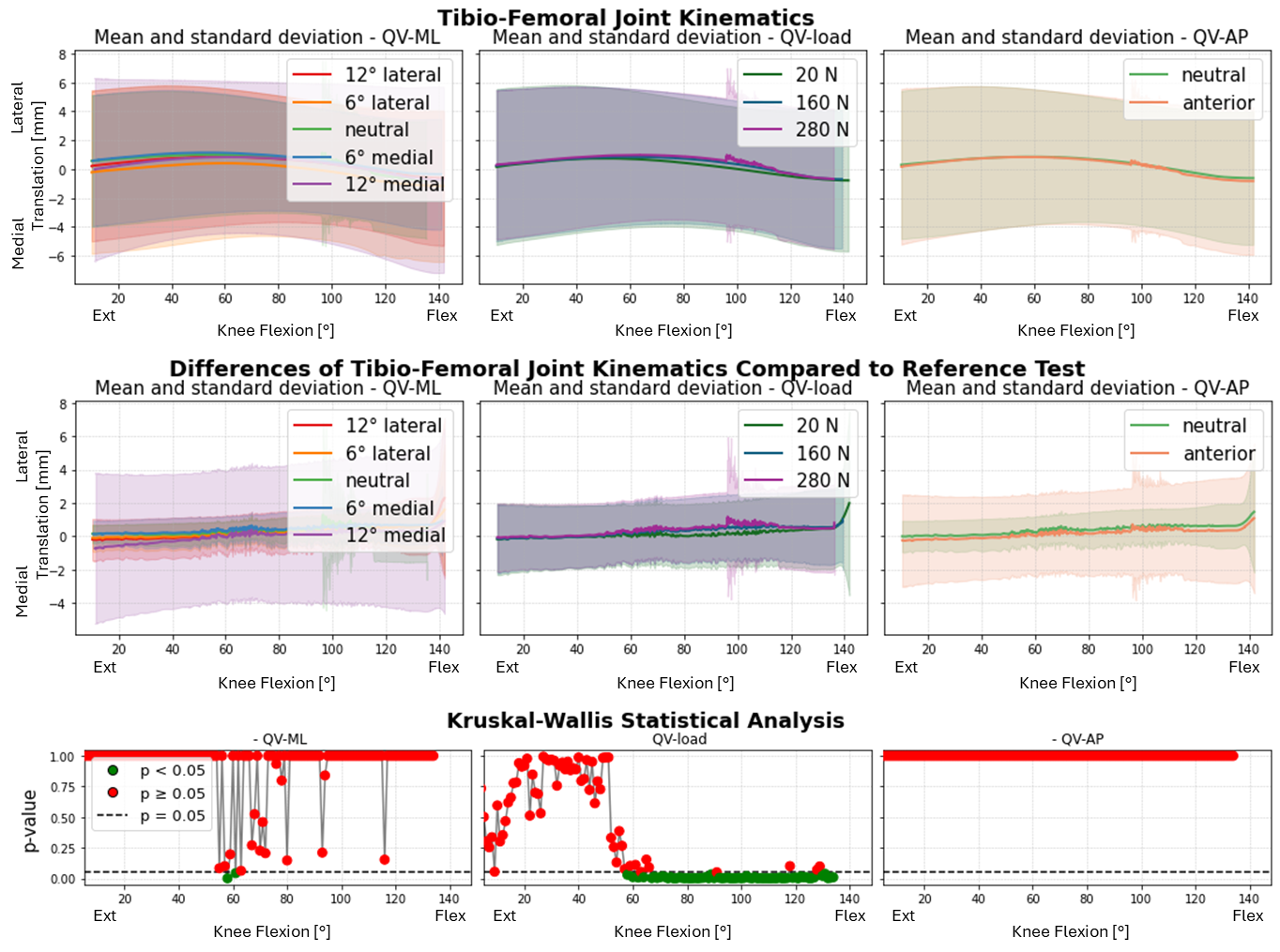


Figure S2_6: Medial-Lateral translation of the femur after TKA. TOP: absolute values as a function of knee flexion angle (the median and standard deviation between 12 specimens are plotted). CENTER: differences of all tests compared with the reference test (QVload = 20 N, QVML = neutral, QVAP = posterior). Left shows the difference as a function of QVML, middle QVload, right QVAP. BOTTOM: significance of the differences plotted at the center. The p-value trend is plotted for the three parameters (left QVML, middle QVload, right QVAP); the significant values are highlighted in green (p<0.05), the non significant ones in red (p≥0.05).
