# Supplementary material for: Effect of femoral component design and quadriceps load on patellofemoral kinematics after total knee arthroplasty: an in vitro cadaveric study
Source: Knee Surg Relat Res. 2026 Feb 26;38:9. doi: 10.1186/s43019-026-00308-6 (PMC12937521; doi:10.1186/s43019-026-00308-6)
Supplement: Supplementary file 4 — Supplementary material 4. [file 43019_2026_308_MOESM4_ESM.docx]

# SUPPLEMENTARY MATERIALS

Appendix to the paper:

Effect of Femoral Component Design and Quadriceps Load on Patellofemoral Kinematics After Total Knee Arthroplasty: An In Vitro Cadaveric Study

## **SUPPLEMENTARY MATERIAL #4**

### **Single specimen kinematic: before and after TKA with patella-friendly femoral component**

To provide an intuitive, detailed illustration of quadriceps-induced kinematic effects in native and implanted knees with patella-friendly design implants, this section focuses on one representative specimen (Specimen #2 with patella-friendly femoral component). For each quadriceps parameter (QV_ML_, QV_AP_, and QV_load_), motion curves are shown for all six patellofemoral kinematic components before and after TKA.

*
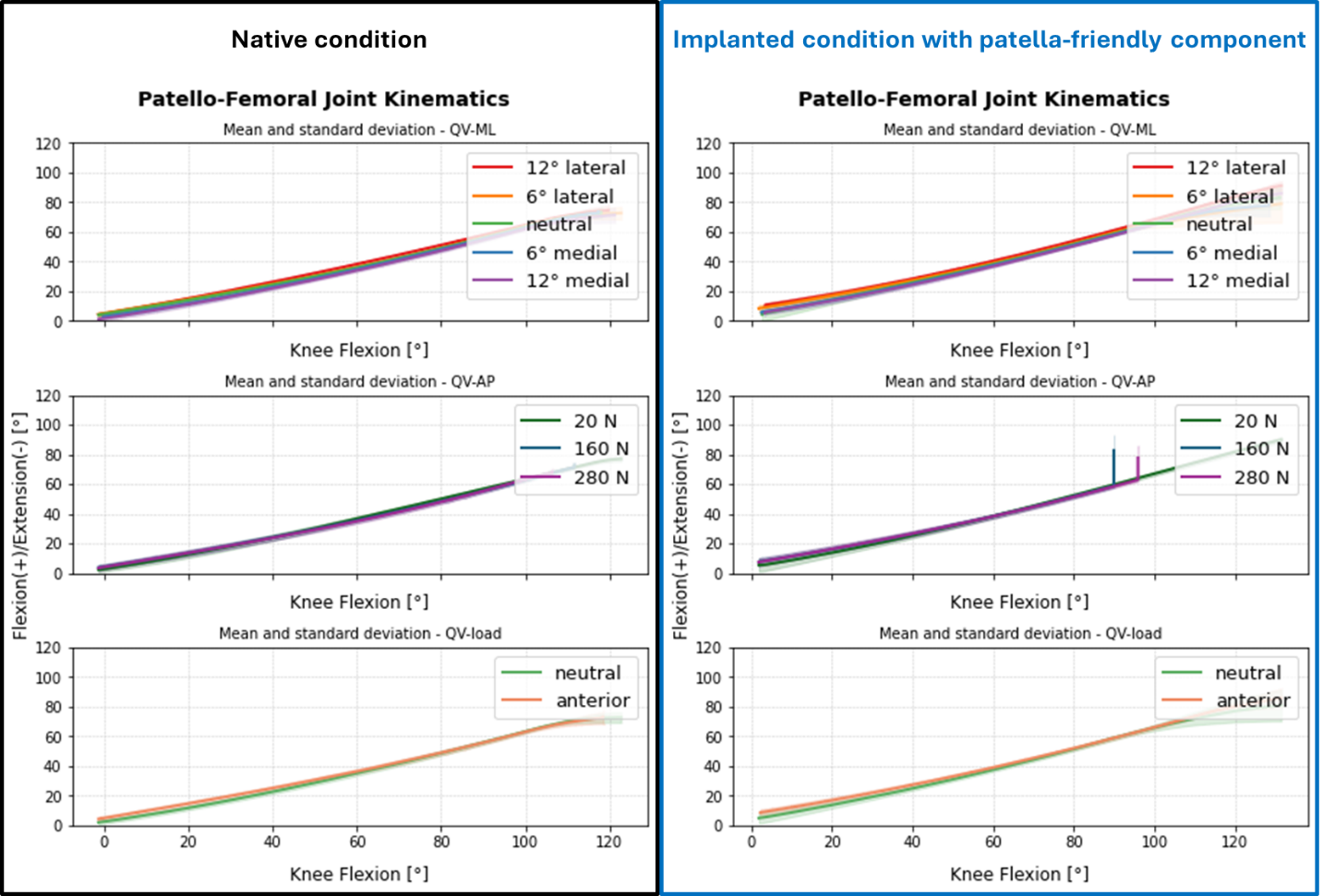
*

Figure S4_1 Flexion/Extension rotation of one specimen (#2) with respect to the knee flexion for all the tests, grouped by QV-ML in the top, QV-load in the middle and QV-AP in the bottom. On the left, in the black box, the native kinematics; on the right, in the blue box, the implanted kinematics with patella-friendly femoral component.


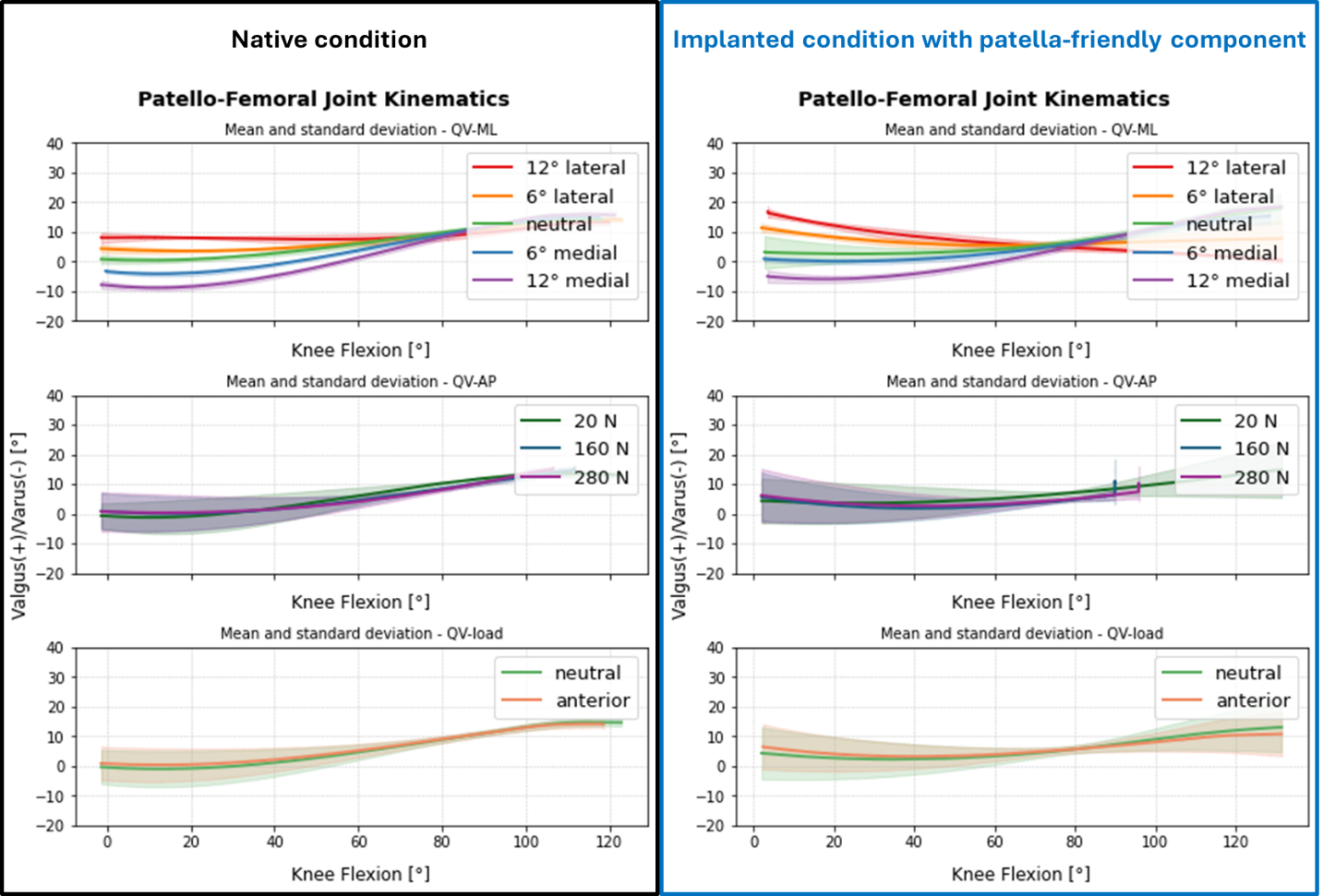


Figure S4_2 Valgus/varus rotation of one specimen (#2) with respect to the knee flexion for all the tests, grouped by QV-ML in the top, QV-load in the middle and QV-AP in the bottom. On the left, in the black box, the native kinematics; on the right, in the blue box, the implanted kinematics with patella-friendly femoral component.


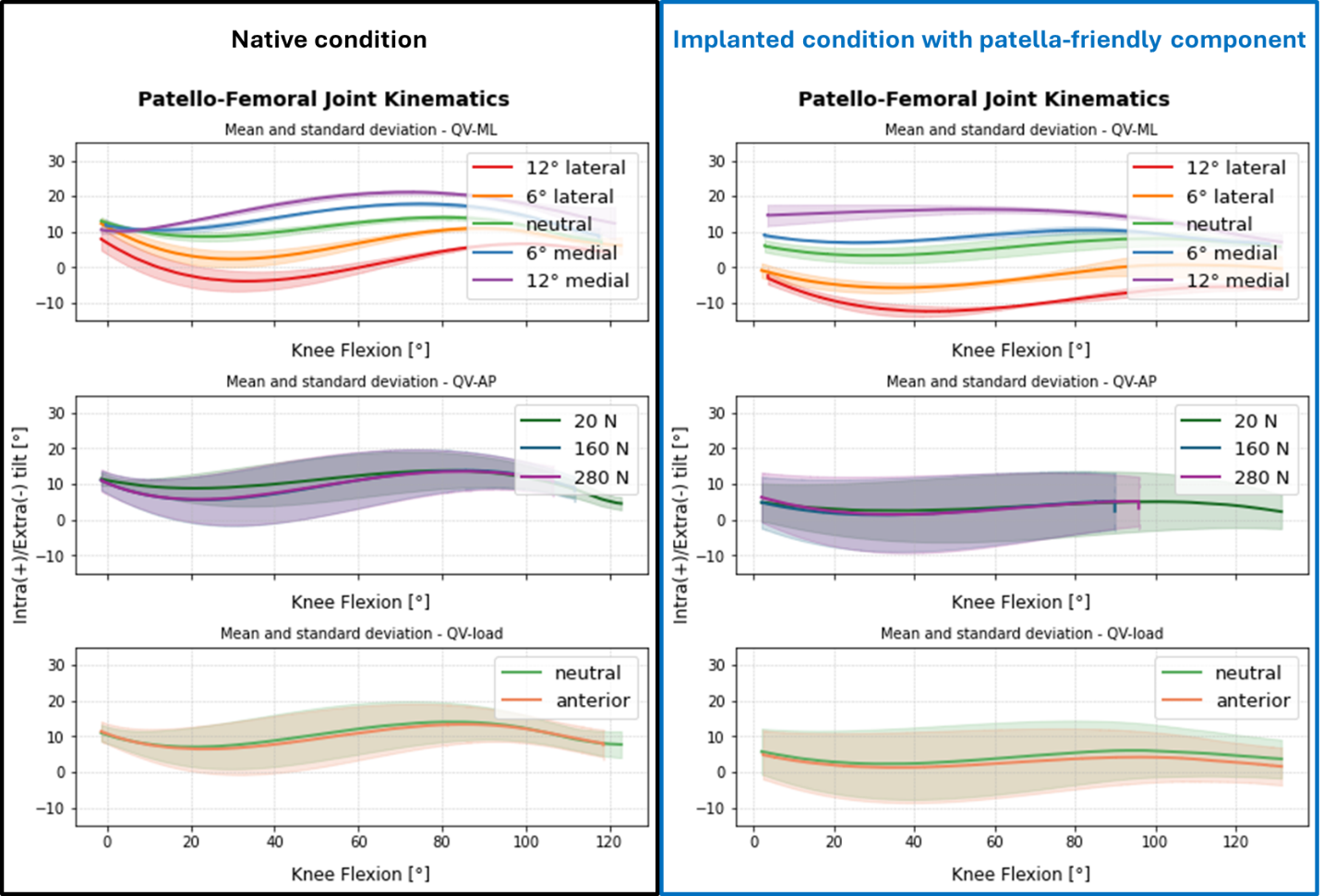


Figure S4_3 Internal/external tilt of one specimen (#2) with respect to the knee flexion for all the tests, grouped by QV-ML in the top, QV-load in the middle and QV-AP in the bottom. On the left, in the black box, the native kinematics; on the right, in the blue box, the implanted kinematics with patella-friendly femoral component.


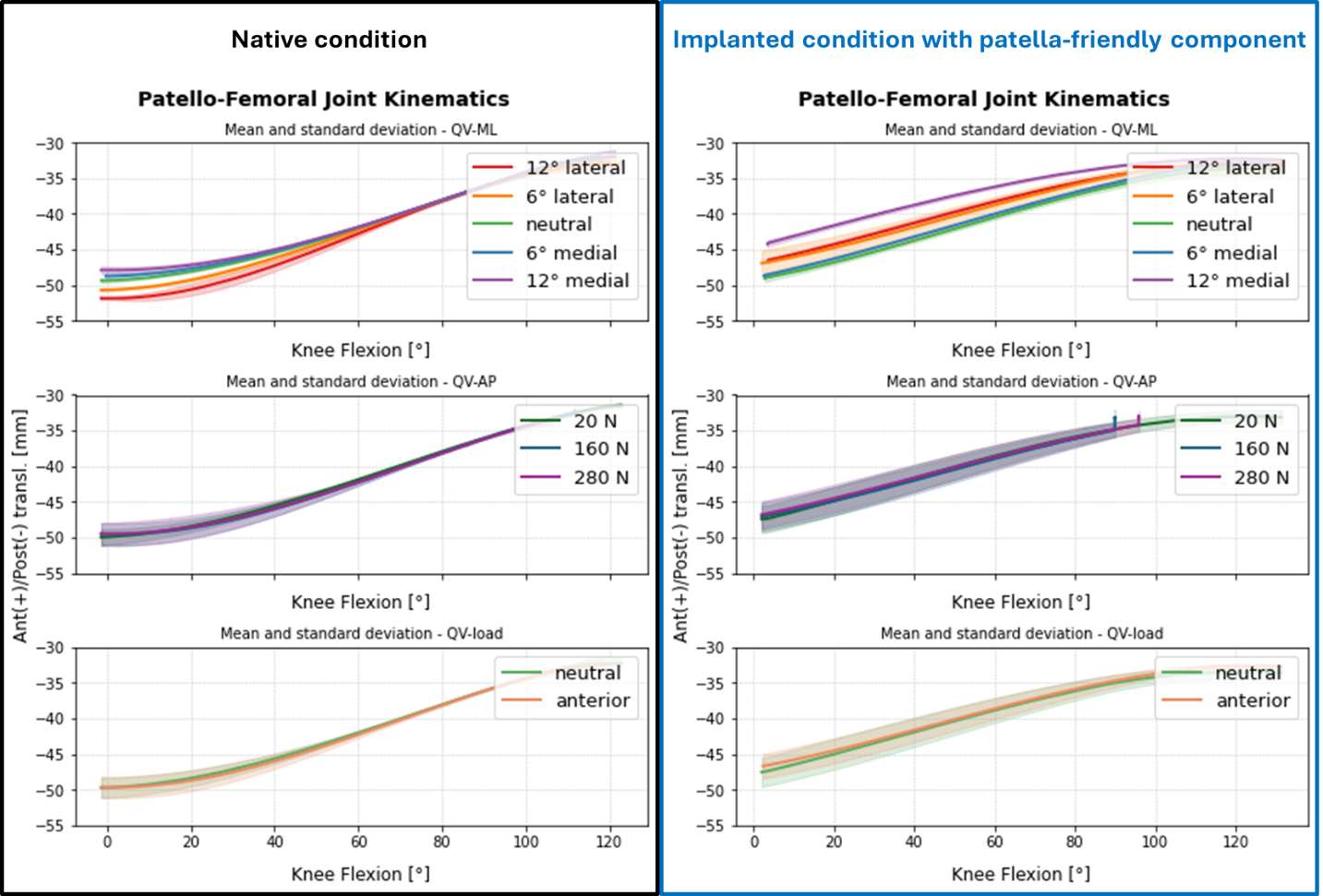


Figure S4_4 Anterior/posterior translation of one specimen (#2) with respect to the knee flexion for all the tests, grouped by QV-ML in the top, QV-load in the middle and QV-AP in the bottom. On the left, in the black box, the native kinematics; on the blue, in the blue box, the implanted kinematics with patella-friendly femoral component.


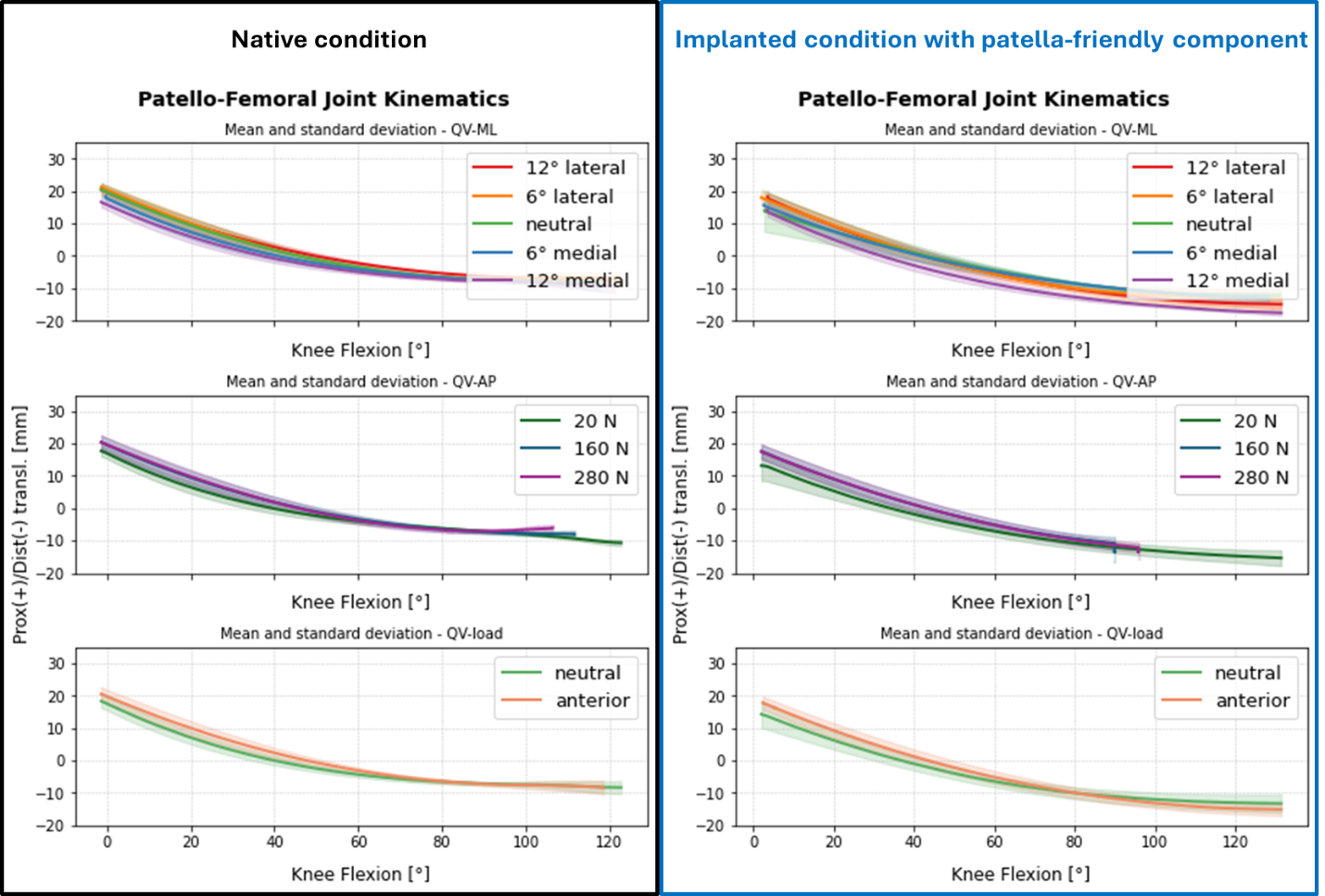


Figure S4_5 Proximal/distal translation of one specimen (#2) with respect to the knee flexion for all the tests, grouped by QV-ML in the top, QV-load in the middle and QV-AP in the bottom. On the left, in the black box, the native kinematics; on the right, in the blue box, the implanted kinematics with patella-friendly femoral component.


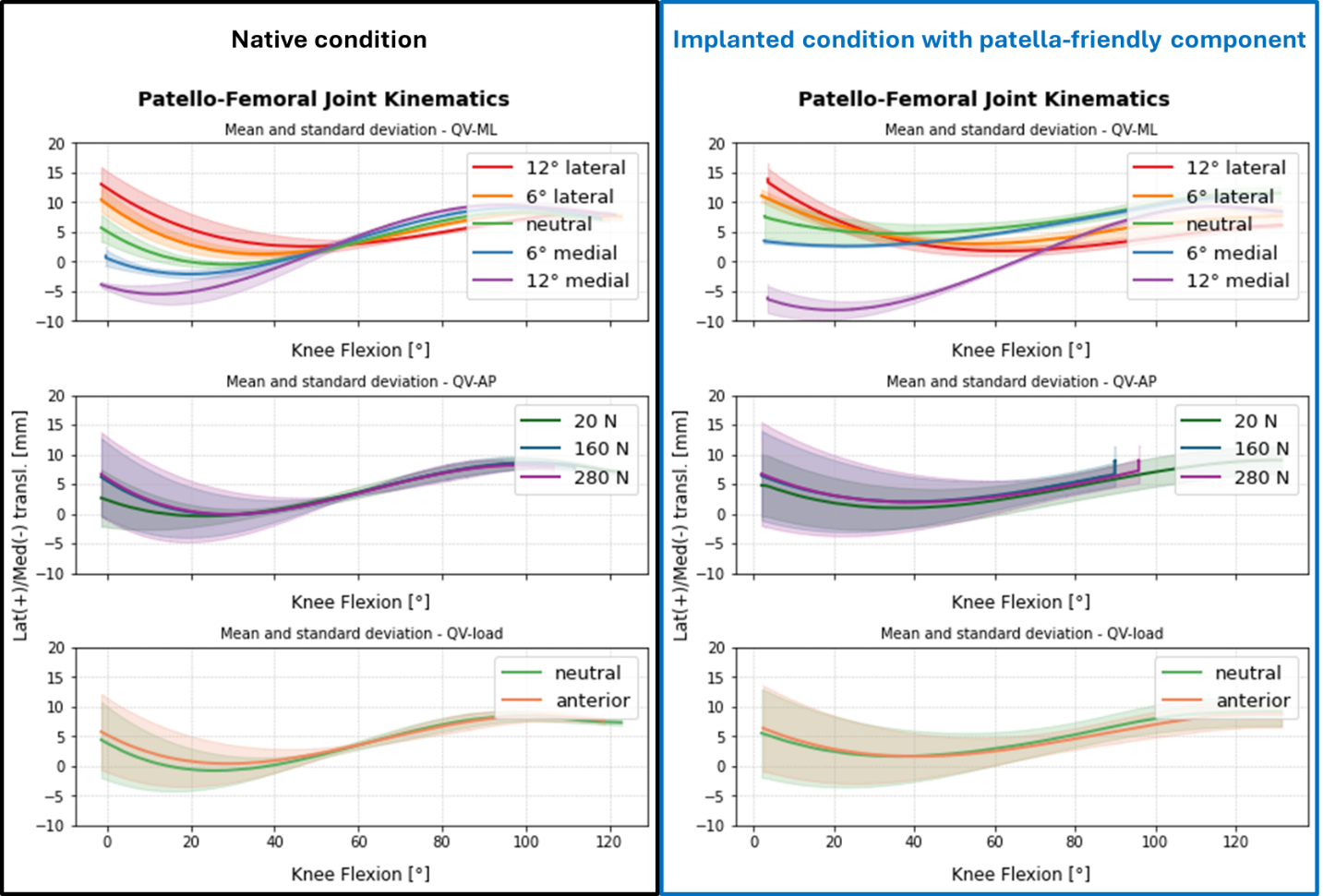


Figure S4_6 Lateral/medial translation of one specimen (#2) with respect to the knee flexion for all the tests, grouped by QV-ML in the top, QV-load in the middle and QV-AP in the bottom. On the left, in the black box, the native kinematics; on the right, in the blue box, the implanted kinematics with patella-friendly femoral component.
